# Biofabrication of zinc-reinforced PLA scaffolds by FDM for bone tissue engineering

**DOI:** 10.1093/rb/rbaf123

**Published:** 2025-12-03

**Authors:** Sahar Halevi, Noam Ribak, Avia Lavon, Sivan Hazan, Galit Katarivas Levy

**Affiliations:** Department of Biomedical Engineering, Ben-Gurion University of the Negev, Beer-Sheva 8499000, Israel; Department of Biomedical Engineering, Ben-Gurion University of the Negev, Beer-Sheva 8499000, Israel; Department of Biomedical Engineering, Ben-Gurion University of the Negev, Beer-Sheva 8499000, Israel; Department of Biomedical Engineering, Ben-Gurion University of the Negev, Beer-Sheva 8499000, Israel; Department of Biomedical Engineering, Ben-Gurion University of the Negev, Beer-Sheva 8499000, Israel

**Keywords:** poly(lactic acid), zinc-reinforced composites, biodegradable scaffolds, fused deposition modelling, bone tissue engineering

## Abstract

The development of biodegradable scaffolds with improved mechanical and biological performance is a pressing challenge in bone tissue engineering. Poly(lactic acid) (PLA), widely used in fused deposition modelling (FDM) due to its processability and biocompatibility, lacks sufficient bioactivity and strength for demanding applications. In this study, we fabricated and evaluated zinc-reinforced PLA composite filaments (5–30 wt% Zn) via melt extrusion and FDM to define a practical printability window and establish process-structure-function relationships aimed at enhancing osteointegration and stability. Microstructure, density, and crystallinity were characterized by optical microscopy/scanning electron microscope, Archimedes’ principle, X-ray diffraction, and differential scanning calorimetry. Mechanical performance was quantified by tensile testing of standardized samples and compression of gyroid lattices. *In vitro* performance was evaluated using human osteoblasts through viability assays, adhesion quantification, and Alizarin Red S staining. Composites incorporating ≤10 wt% Zn showed uniform particle dispersion without impairing printability. Zn10 (10 wt% Zn) recovered PLA-like tensile strength with the highest strain-at-fracture among groups and exhibited significantly higher compressive strength and modulus than PLA and Zn5 (5 wt% Zn). All groups were found to be non-cytotoxic (∼100% viability) and supported osteoblast adhesion. Notably, zinc-containing scaffolds promoted significantly higher calcium deposition after 28 days, demonstrating enhanced late-stage osteogenic differentiation. These findings demonstrate that low-level Zn reinforcement can improve both structural integrity and biological performance of PLA-based scaffolds, supporting Zn-reinforced PLA as a scalable, extrusion-ready platform for the biofabrication of patient-specific bone-regenerative implants.

## Graphical abstract

**Figure:**
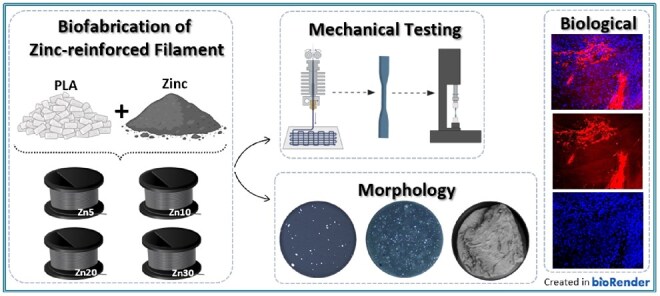

## Introduction

Bone tissue engineering requires the development of biomaterials that are not only biodegradable and biocompatible but also possess sufficient mechanical integrity and bioactivity to support osteogenesis [1]. Among available polymers, poly(lactic acid) (PLA) has garnered significant attention due to its renewability, biodegradability, and approval for biomedical use [2–4]. PLA is classified as “Generally Recognized as Safe” (GRAS) by the US FDA and is commonly employed in drug delivery systems, tissue fixation devices, and temporary implants [5, 6]. Upon degradation, PLA converts into lactic acid, a metabolite readily processed by the human body, making it well-suited for temporary implants and tissue scaffolds [3, 7, 8]. Structurally, PLA is a semi-crystalline polymer that exhibits a mixture of crystalline and amorphous regions. The crystalline phase can exist in two primary polymorphs: the more ordered α-form and the less-ordered α′-form. Formation of these phases depends on thermal history, processing conditions, and molecular stereoregularity [9–12]. The degree and nature of crystallinity significantly influence PLA’s mechanical performance, degradation rate, and thermal stability. Specifically, increasing the crystalline content, especially the α-form, generally improves stiffness and thermal resistance, though it may compromise biodegradability [13].

Additive manufacturing technologies have transformed the fabrication of complex, patient-specific medical devices and implants [14]. Among these, fused deposition modelling (FDM), a form of material extrusion as defined by ISO/ASTM 52900:2021 [15], is one of the most accessible and widely adopted methods due to its low cost, material versatility, and ease of customization [16, 17]. FDM involves the layer-by-layer extrusion of thermoplastic filaments through a heated nozzle to construct 3D objects based on digital models. PLA’s low glass transition temperature (*T*_g_ ≈ 60°C) and good printability have made it the material of choice for FDM-based biomedical scaffolds [18, 19]. Due to these properties, PLA is one of the most popular thermoplastic polymers in FDM printing.

Despite these advantages, neat PLA has inherent limitations for load-bearing applications, including brittleness, limited degradation control, hydrophobicity, and poor osteoinductivity [4, 20–22]. Reinforcement with bioactive metallic fillers such as magnesium (Mg), titanium (Ti), or zinc (Zn) has been proposed to address these shortcomings. Representative studies on PLA composites with metallic fillers are summarized in Table S1 [4, 5, 8, 20, 23–31]. These metallic fillers can significantly increase the load-bearing capacity and stiffness of PLA-based scaffolds while also providing biological benefits, such as promoting osteoblast adhesion, stimulating osteogenic differentiation, and exhibiting antibacterial properties, through the controlled release of bioactive metal ions [3, 8, 24]. Nevertheless, the success of such reinforcement strategies depends critically on filler type, size, dispersion, interfacial adhesion, and concentration. Improperly dispersed fillers may create agglomerates and act as stress concentrators, weakening the matrix and reducing mechanical reliability [3, 6, 32, 33].

**Figure 1.**
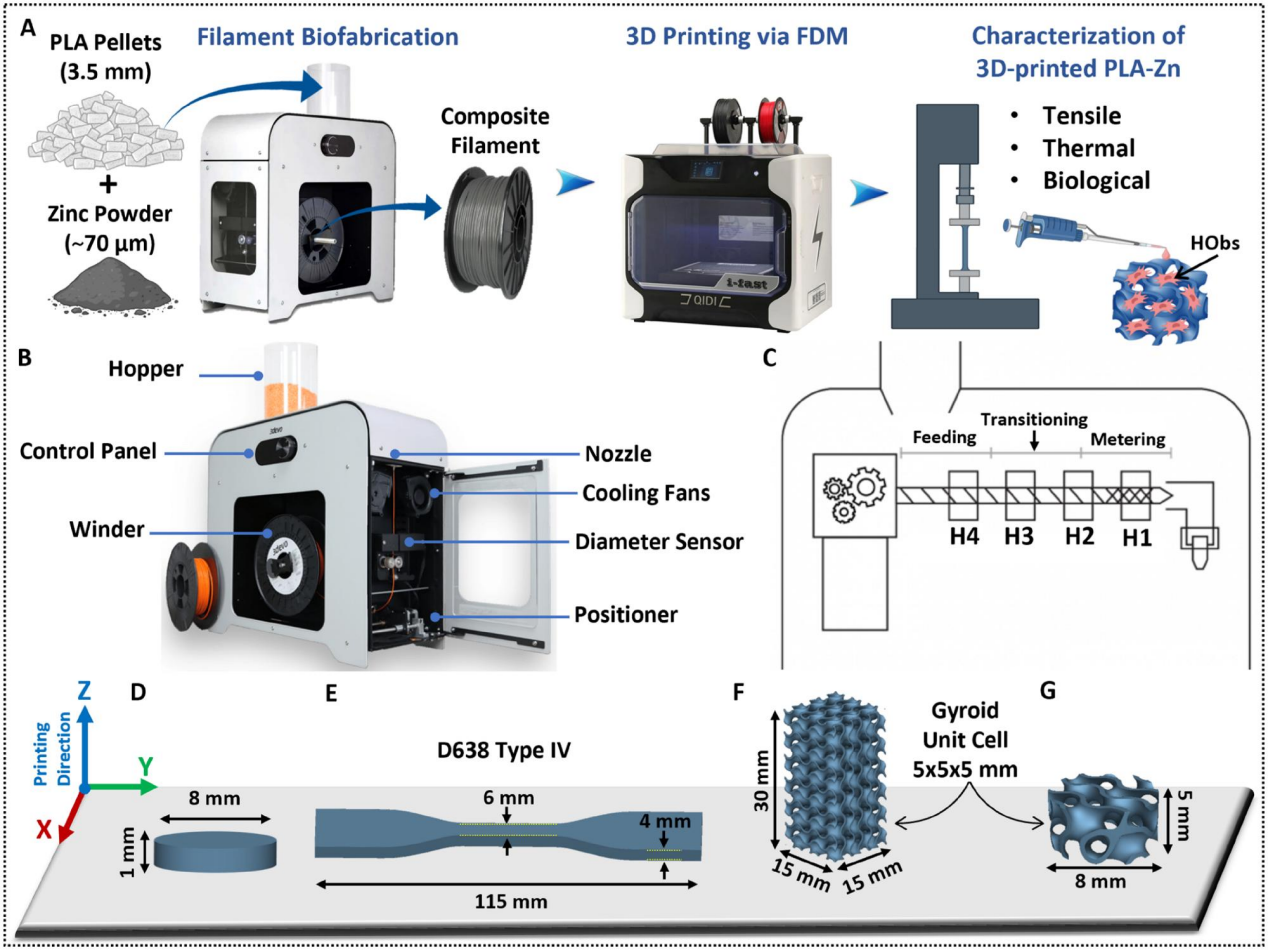
(**A**) Schematic overview of filament production, printing and characterization workflow. (**B**) Description of the filament extruder and its components. (**C**) Schematic of the composer 450 heating zones. (**D**) Designed models for evaluating particle distribution after FDM, (**E**) ASTM type IV tensile test specimen, (**F**) The designed compression model with gyroid lattice structure, 5x5x5 mm unit cell and 0.8 mm wall thickness, (**G**) The designed scaffold for *in vitro* tests. Some of the images were created in nTopology and BioRender.com.

This study investigates the extrusion, 3D printing, and biological evaluation of novel Zn-reinforced PLA filaments to generate biodegradable scaffolds. Zinc was selected as the reinforcing phase because it is an essential trace element in bone physiology (enzymatic regulation, osteogenesis, immune modulation), exhibits osteogenic and antibacterial activity, and can augment scaffold functionality via controlled ion release, while degrading at a moderate rate without gas evolution [4, 5, 34–38]. By systematically evaluating zinc concentrations (5–30 wt%) and their effects on filament and 3D-printed samples quality, homogeneity, microstructure, crystallinity, mechanical performance, and *in vitro* osteogenesis potential (Figure 1A), we aim to optimal formulations for translational bone scaffold applications. Unlike earlier reports that emphasized composition-property snapshots, our study integrates a broad 5–30 wt% Zn range to establish a practical printability window, and quantitatively links processing to dispersion/porosity, crystallinity, and mechanical behaviour, as well as time-resolved osteogenesis (28-day mineral deposition) *in vitro*. This process-structure-function mapping provides actionable operating guidance that extends beyond composition alone. The resulting structure-property-function relationships provide a foundation for the scalable biofabrication of next-generation bone-regenerative implants using accessible extrusion-based 3D printing platforms. In doing so, this work advances patient-specific medicine by aligning materials processing windows with clinically practical manufacturing compatible with patient-specific customization. More broadly, it advances the composite materials field by showing how metal-polymer systems can be fine-tuned to the application, balancing processing stability, microstructure, mechanics, and bioactivity, and may thereby reveal emergent materials properties that inform the discovery and optimization of new multifunctional bioactive composites.

## Materials and methods

### Materials

The materials used in this study included granulated PLA 4043D (3devo, Netherlands), with a molecular weight of 110 kDa and a D-isomer content of 4.5–5 wt% [39], and zinc powder (Z1-10027-P, Testbourne Ltd, England) with an average particle size of 70 µm and 99.9% purity. PLA pellets were dried in an oven at 50°C for 4 hours (Ari Levy Ltd, Israel) to remove moisture, then mixed with zinc powder to prepare composite batches containing 5, 10, 20, and 30 wt% Zn. These were labelled Zn5, Zn10, Zn20, and Zn30, respectively.

### Filaments biofabrication via extrusion process

Filament biofabrication was carried out using the Composer 450 Filament Maker (3devo, Netherlands). As illustrated in Figure 1B and C, the device features a hopper for feeding raw materials, a nitride-hardened steel mixing screw housed within a heated barrel, four independently controlled heating zones, a 4 mm nozzle, a dual-fan cooling system for uniform air distribution, an optical diameter sensor, and a puller system to ensure consistent filament dimensions. For neat PLA, the standard settings recommended by the manufacturer were employed. For the zinc-reinforced PLA composite, heating zone temperatures and fan speeds were adjusted as needed during extrusion to maintain a target filament diameter of approximately 1.75 mm, based on visual assessment and diameter feedback from the sensor. The biofabrication process parameters are summarized in Table 1.

**Table 1.** Filaments biofabrication and 3D printing parameters used to manufacture the samples in this study

Filaments biofabrication							
Parameter	Temperatures (°C)				Screw speed (RPM)	Fan speed (%)	Puller speed
	H4	H3	H2	H1			
PLA	170	185	190	170	3.5	80	Auto
Zn5	170	185	185	168	3.5	80	
Zn10	170	183	182	170	3.5	90	
Zn20	160	175	180	162	3.5	85	
Zn30	156	170	175	158	3.5	100	

3D-printing	
Parameter	Value
Extrusion multiplier	1
Nozzle diameter	0.4 mm
Nozzle material	Hardened steel
Infill percentage	100%
Infill geometry	Aligned rectilinear ±45°
Perimeter (outer shell)	2
Printing speed	50 mm⋅s^−1^
First layer speed	20 mm⋅s^−1^
Layer height	0.2 mm
Extrusion width (layer width)	0.42 mm
Seam position	Aligned

### 3D printing

Three-dimensional models were designed using nTopology software (nTopology, New York, USA) that were then exported as STL files. These files were converted into printer-readable GCODE using Qidi Print 6.5.4 (Qidi Tech, WenZhou City, Zhejiang, China). Fabrication was performed using an FDM printer (I-Fast, Qidi Tech, WenZhou City, Zhejiang, China) equipped with a high-temperature dual-nozzle extruder. Prior to printing, filament spools were dried in an oven (Ari Levy Ltd, Israel) at 50°C for 4 hours to minimize moisture content. Test specimens were printed using both neat PLA and Zn-reinforced PLA composite filaments under the printing conditions summarized in Table 1.

### Microstructure characterization

Cross-sectional analysis of the Zn-reinforced PLA filaments was performed using an Axio Observer A1 optical microscope (Zeiss, Germany) and a desktop scanning electron microscope (SEM) (Phenom XL, Thermo Fisher Scientific, Eindhoven, The Netherlands). Filament samples were embedded in VersoCit-2 resin and polished using a LaboPol-2 system (Struers, Denmark) to expose their internal structure. Both bright-field (BF) and dark-field (DF) imaging modes were utilized to assess zinc particle dispersion and porosity. Additionally, small disc-shaped specimens (8 mm diameter, 1 mm height) were 3D-printed and examined under a Revolution Microscope (ECHO, USA) to evaluate zinc particle distribution within the printed matrix. Image analysis was conducted using ImageJ software to quantify the number and distribution of zinc particles within the imaged area [40]. The X-ray diffraction (XRD) investigation of the 3D-printed samples (4 × 19 × 80 mm) was performed using an Empyrean II multipurpose diffractometer (Malvern Panalytical, Malvern, Worcestershire, United Kingdom; Almelo, Netherlands) with a CuKα (1.540 Å) anode at room temperature. XRD data were collected in the angular range where 2θ = 5°–90°. With a scan speed of 0.00846°⋅sec^−1^.

### Density measurement

The density of the filaments was determined using Archimedes’ principle. To ensure reproducibility, five specimens (*n =* 5) from each filament type were measured under identical conditions (Figure 1D). The experimental density (ρ) was calculated using the equation:


$$\rho =\frac{W_{a}}{W_{a}-W_{w}}\cdot \rho_{w},$$

where *W*_a_ is the weight of the sample in air, *W*_w_ is the weight in water, and ρ_w_ is the density of water, taken as 0.9982 g⋅cm^−3^ at a room temperature of 20°C [41]. The theoretical densities of the Zn-reinforced PLA composites were calculated using the rule-of-mixture equation [42]:


$$\rho_{\text{Theoretical}}=\frac{1}{\left(W_{\text{Zn}}/\rho_{\text{Zn}})+(W_{\text{PLA}}/\rho_{\text{PLA}}\right)},$$

where *W* represents the weight fraction of each material in the composite, based on molecular weights, and ρ denotes the material densities: ρ_PLA_ = 1.24 g⋅cm^−3^ [43, 44] and ρ_Zn_ =7.14 g⋅cm^−3^ [5, 45–47].

### Differential scanning calorimetry

Differential scanning calorimetry (DSC) analysis of PLA and Zn-reinforced PLA composites was performed using a DSC 25 analyser (TA Instruments, CA, USA). Approximately 10 mg of each sample was analysed. The thermal program involved heating from 25°C to 200°C, cooling back to 25°C, and reheating to 200°C. All heating and cooling stages were conducted at a rate of 5 °C⋅min^−1^. Thermal transitions, including glass transition temperature (*T*_g_), cold crystallization temperature (*T*_c_), and melting temperature (*T*_m_), were identified from the heating scans. *T*_g_ was determined as the midpoint of the baseline shift corresponding to the glass transition. The degree of crystallinity (*X*_c_) was calculated using the following equation [20, 48]:


$${\;X}_{c}(\%)=\frac{\Delta H_{m}-\Delta H_{c}}{\Delta H_{m}{}^{\circ}}\cdot \frac{100\%}{w},$$

where Δ*H*_m_ is the enthalpy of fusion, Δ*H*_c_ is the enthalpy of cold crystallization, Δ*H*_m_° is the enthalpy of fusion for 100% crystalline PLA (93 J⋅g^−1^), and *w* is the weight fraction of PLA in the composite [49, 50].

### Mechanical testing

Mechanical testing was conducted using a Lloyd LRX Plus universal testing machine (Ametek, Inc., PA, USA) with a 5 kN load cell. Tensile test was performed according to ASTM D638 [51] with a strain rate of 5 mm⋅min^−1^ until sample failure. Type IV specimens (Figure 1E) were printed in a flat orientation, as this configuration provides an optimal balance of strength, dimensional accuracy, and consistency, and enables analysis of fracture behaviour [52]. Compression test was performed according to ASTM E9-19 [53] and ISO 13314:2011 [54], with a compressive rate of 5 mm⋅min^−1^ until the sample fails or reaches the maximum load. Compression specimens were designed and 3D-printed with dimensions of 15 × 15 × 30 mm (Figure 1F), featuring a gyroid lattice structure with a unit cell dimension of 5 × 5 × 5 mm and a wall thickness of 0.8 mm using nTopology. To ensure data reproducibility, five specimens (*n = 5*) were tested under identical processing conditions. Tensile strain was measured from the loading cell displacement and recorded using an Epsilon 3542 axial extensometer (Epsilon Technologies, Jackson, WY, USA). The elastic modulus, representing material stiffness, was calculated from the slope of the stress–strain curve within the linear elastic region [40, 55]. Ultimate strengths were defined as the maximum stress recorded immediately after yield on the stress–strain curve [45, 56].

### Fractography morphology

The fracture surface of the tensile samples was analysed using a desktop SEM (Phenom XL, Thermo Fisher Scientific, Eindhoven, The Netherlands). Acceleration voltage was set to 10 kV, and a CeB6 electron source was used. The fracture surface was coated with a 10 nm thick layer of gold prior to SEM inspection using a sputter coater (Q150TS Plus, Quorum Technologies, Sacramento, CA, USA).

### *In vitro* cytotoxicity, biocompatibility and osteointegration capability

The *in vitro* evaluation included both indirect and direct assays to assess the cytotoxicity, biocompatibility, and osteointegration potential of the Zn-reinforced PLA composites, in accordance with ISO 10993-5 standards for the biological evaluation of medical devices [57]. Human osteoblasts (HObs) were used under standard culture conditions (37°C, 5% CO_2_). Cells were maintained in minimum essential medium alpha (MEM-α; Sartorius, Germany) supplemented with 10% fetal bovine serum and 1% Pen-Strep-Ampho-B solution (containing 10 000 units⋅mL^−1^ penicillin, 10 mg⋅mL^−1^ streptomycin, and 0.025 mg⋅mL^−1^ amphotericin B). Disc-shaped scaffolds (10 mm diameter, 5 mm height) featuring a gyroid lattice structure (unit cell: 5 × 5 × 5 mm) were fabricated using neat PLA and Zn-reinforced PLA composites (Figure 1G). To ensure reproducibility, all biological assays were performed in triplicate across three independent experiments (*n = 3* per group). Prior to cell seeding, all scaffolds were sterilized by immersion in 0.3% sodium hypochlorite for 15 minutes, followed by thorough rinsing with sterile deionized water (dH_2_O) [40, 58].

#### Cytotoxicity analysis via indirect cell viability assay

Cytotoxicity was evaluated using an indirect extract method. Zn-reinforced PLA composite scaffolds, along with neat PLA samples, were incubated in a complete culture medium for 24 hours to obtain extract media. In parallel, HObs were seeded in 96-well plates at a density of 1 × 10^4^ cells per well and incubated for 24 hours to allow cell attachment. After the initial incubation, the conditioned media (extracts) from each scaffold group were collected and used to replace the culture medium in the seeded wells. Cells cultured in fresh medium served as the negative control, while medium containing 10% dimethyl sulfoxide (DMSO) was used as a positive control for cytotoxicity. Cell viability was assessed after 24 and 48 hours of exposure using the Cell Counting Kit-8 (CCK-8). Optical density (OD) was measured at 450 nm using a BioTek Epoch Microplate Spectrophotometer (Agilent, Santa Clara, USA). Cell viability (%) was calculated using the following equation [37, 59]:


$$\text{Cell}\;v\text{iability}\;(\%)=\frac{OD_{\text{sample}}}{OD_{\text{control}}}\times 100\%,$$

where OD_sample_ represents the absorbance of cells treated with scaffold extracts, and OD_control_ corresponds to the absorbance of the negative control group.

#### Direct cell viability assay

Successful osseointegration of a scaffold requires the migration, attachment, and differentiation of osteoprogenitor cells into mature osteoblasts at the implant surface [60]. A direct cell viability assay was performed to evaluate the initial osteointegration potential of the Zn-reinforced PLA scaffolds. A 100-μL droplet containing 2 × 10^5^ human osteoblasts (HObs) suspended in culture medium was seeded onto each scaffold and incubated under standard culture conditions (37°C, 5% CO_2_) for 6 hours to promote cell attachment. After this initial incubation, each scaffold was supplied with 1 mL of fresh culture medium and maintained for 28 days under identical conditions. On day 7, osteogenic differentiation was induced by supplementing the medium with 10 nM dexamethasone (D2915, Sigma, MA, USA) and 10 mM β-glycerophosphate (10424701, Fisher Scientific, PA, USA). The medium was replaced every two days. Calcium deposition by osteoblasts was evaluated on days 14, 21, and 28 using Alizarin Red S staining (ARS; Holland Moran, Israel), which detects calcium-rich extracellular matrix deposits. Samples were first rinsed with phosphate-buffered saline, fixed in 4% (v/v) formaldehyde for 30 minutes at room temperature, and then washed with sterile deionized water (dH_2_O). Staining was performed by immersing the samples in 1 mL of 2% (w/v) Alizarin Red solution (pH adjusted to 4.2 with 0.5% ammonium hydroxide) for 15 minutes. After staining, the samples were thoroughly washed with dH_2_O to remove unbound dye. For quantitative cell counting to assess cell adhesion and proliferation, nuclei were counterstained with 4′,6-diamidino-2-phenylindole (DAPI), and fluorescence imaging was performed using the ECHO Revolution fluorescence microscope (Echo, San Diego, CA, USA) (DAPI: 460 nm, ARS: 580 nm). ImageJ software was used to quantify the number of adherent cells and assess mineralization within defined image areas.

### Statistical analyses

A one-way analysis of variance (ANOVA) was performed to determine whether the measurement groups had statistically significant differences in their means. Pairwise comparisons were conducted using Tukey’s honest significant difference test, with statistical significance set at *P* < 0.05. The significance levels are denoted as follows: “ns” indicates no significant difference, *P < 0.05, **P < 0.01, ***P < 0.001, and ****P < 0.0001 [45].

## Results and discussion

### Filament extrusion

**Figure 2.**
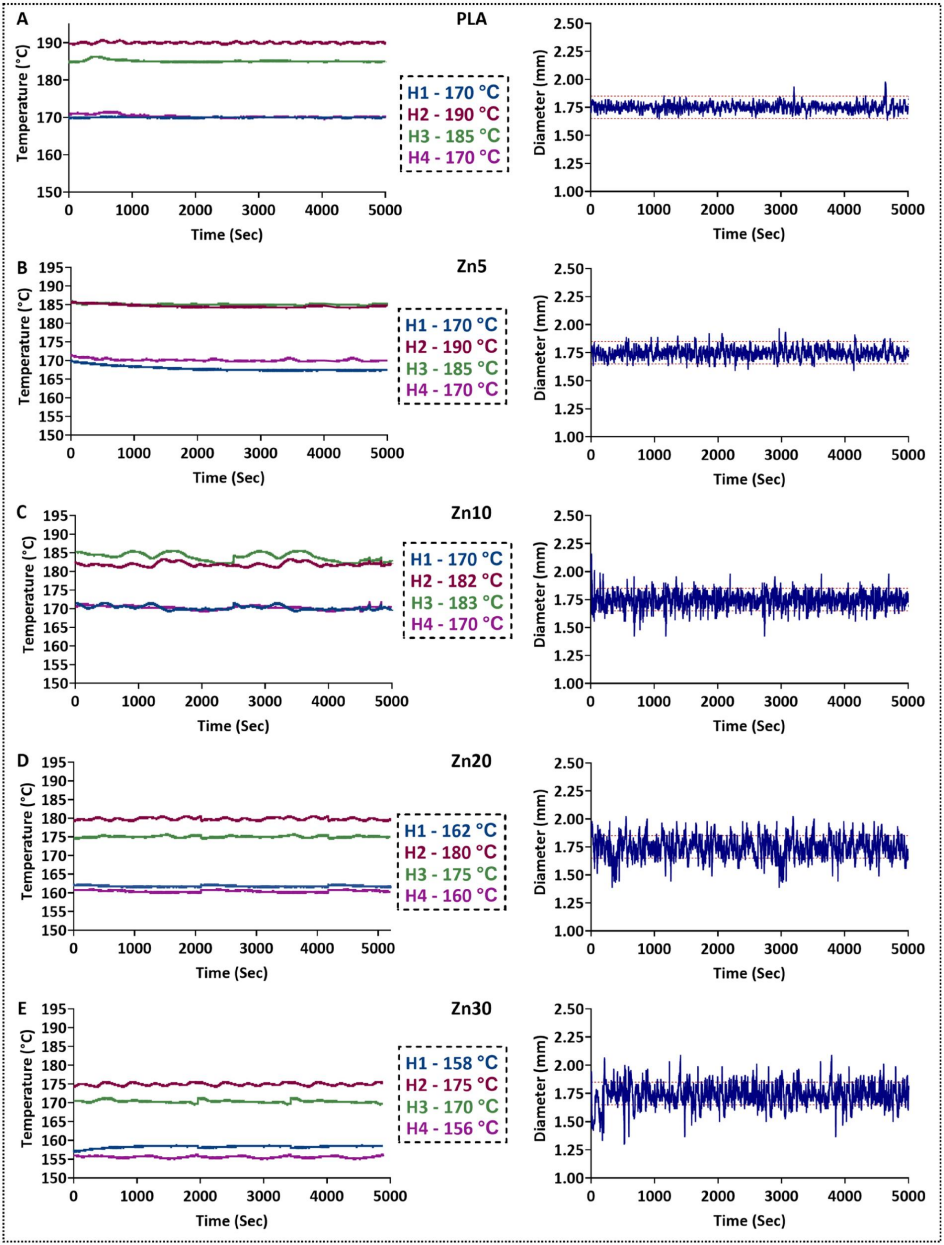
Extrusion data showing the temperatures of the four heating zones and corresponding filament diameter measurements over fabrication time for (**A**) PLA, (**B**) Zn5, (**C**) Zn10, (**D**) Zn20, and (**E**) Zn30. H4 corresponds to the heating zone located closest to the hopper, while H1 is positioned nearest the nozzle. The target filament diameter was set at 1.75 mm, with acceptable upper and lower tolerance limits of 1.85 and 1.65 mm, respectively (indicated by red dashed lines).

Figure 2 presents the extrusion data for PLA and the Zn-reinforced composite filaments, as recorded by the 3devo DevoVision monitoring software. The graphs display the temperature profiles for the four extruder heating zones (H1–H4) and the corresponding real-time filament diameter measurements. The red dashed lines on the diameter plots indicate the acceptable tolerance range of 1.65–1.85 mm. For neat PLA (Figure 2A), the filament diameter remained stable throughout the extrusion process, averaging 1.75 ± 0.1 mm. A similar trend was observed for the Zn5 and Zn10 composites (Figure 2B and C), indicating consistent extrusion behaviour at low filler content. However, with increased zinc content (20% and 30%; Figure 2D and E), significant fluctuations in filament diameter were recorded, often exceeding the acceptable range. These variations are consistent with changes in melt viscosity and heat-transfer characteristics associated with zinc incorporation. In particular, the addition of zinc may lower the effective melt viscosity, suggesting the need for reduced extrusion temperatures to maintain flow stability and cooling efficiency [4, 5]. Furthermore, zinc’s high thermal conductivity (113 W·m^−1^·K^−1^) [61] compared to PLA (0.193 W·m^−1^·K^−1^) [62] significantly altered the thermal dynamics of the extrusion process. The increased heat transfer in PLA-Zn mixtures reduced PLA thermal stability, leading to earlier viscosity reduction near the nozzle [63]. As a result, the extrudate reached the puller mechanism before adequate cooling, particularly at higher zinc concentrations, causing irregular filament diameters. To address these challenges, extrusion parameters were optimized based on the baseline settings recommended by the extruder manufacturer for neat PLA [64]. A reduction in the heating zone temperatures was essential for achieving precise control over the cooling process and final diameter of the Zn-reinforced PLA composites; for the Zn30 formulation, this reduction reached up to 14°C in H4 and 12°C in H1. Without such adjustments, the filament would not solidify sufficiently before reaching the spool, resulting in dimensional inconsistencies. Maintaining a consistent filament diameter is essential as deviations can lead to feeding issues as oversized filament may jam or increase friction in the drive gear of the extruder, while undersized filament may slip due to insufficient grip and result in under-extrusion [65]. As zinc concentration increased, fine-tuning the extrusion conditions became increasingly complex, highlighting the processing limitations and sensitivity of the system at higher filler loadings.

### Microstructure characterization and density

**Figure 3.**
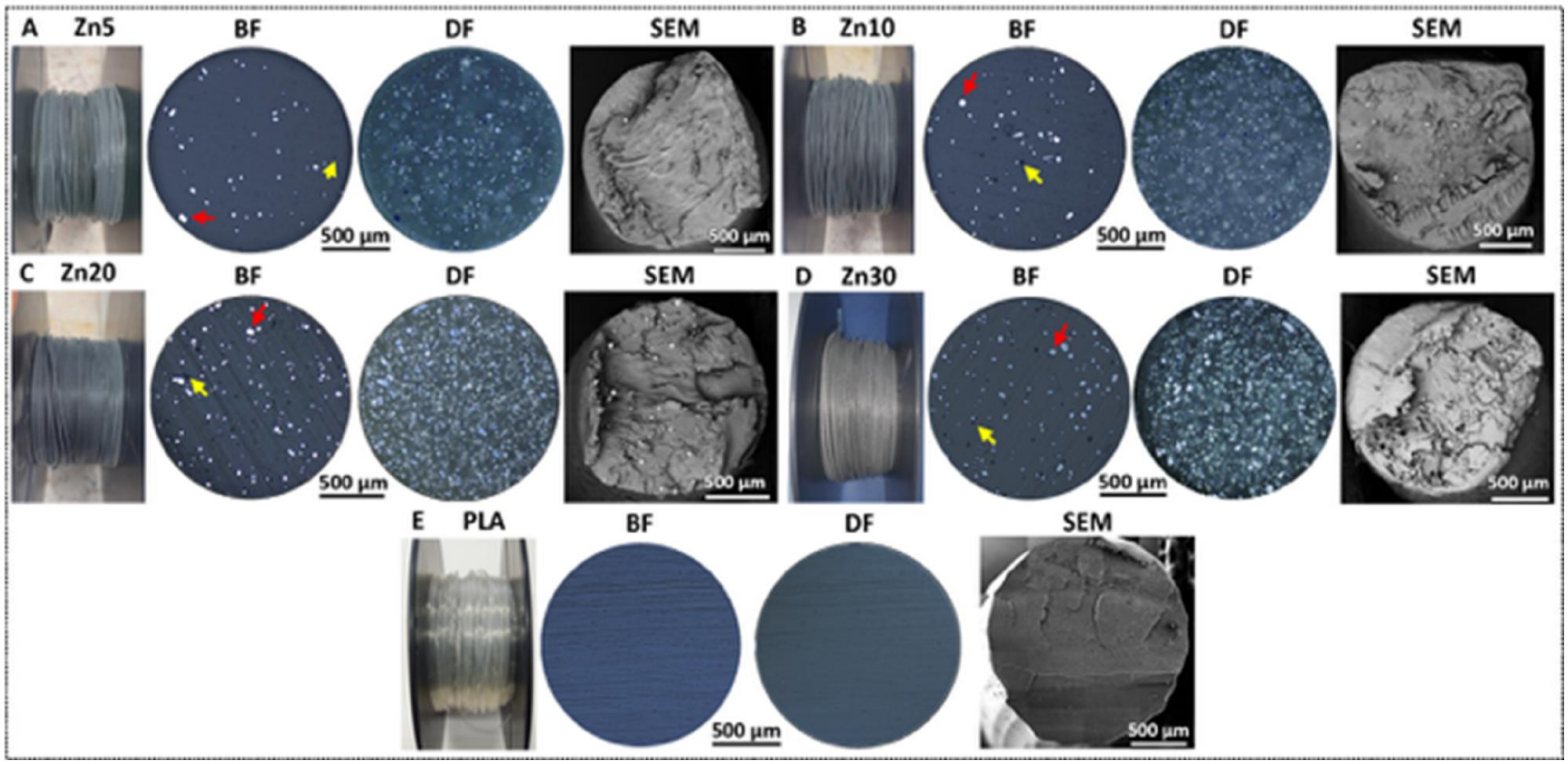
Cross-sectional micrographs of Zn-reinforced PLA and PLA filaments were acquired using bright-field (BF) and dark-field (DF) optical microscopy, as well as scanning electron microscopy (SEM). Red arrows mark the zinc particles, while yellow arrows mark the presence of pores. Images are shown for (**A**) Zn5, (**B**) Zn10, (**C**) Zn20, (**D**) Zn30, and (**E**) neat PLA.

A uniform distribution of metallic fillers in a polymer matrix is crucial for achieving homogeneous physical and mechanical properties in the composite. This is particularly important in the context of bone-regenerative implants, where consistent mechanical behaviour is critical, even under low mechanical loading conditions [66]. Figure 3 presents BF, DF and SEM cross-sectional microscopic images of PLA and Zn-reinforced PLA filaments. BF and SEM images show zinc particles are well integrated within the PLA matrix across all compositions, with no evident signs of particle pull-out. However, an increase in microporosity was observed with rising zinc content. While neat PLA and Zn5 exhibited minimal porosity, Zn10, Zn20, and especially Zn30 showed a marked increase in both the number and size of micropores.

The formation of these voids is likely due to a combination of factors occurring during the extrusion and cooling processes. These factors include changes in the orientation of zinc particles during extrusion, as well as gas entrapment, where trapped gas layers prevent the polymer matrix from completely filling the gaps between particles [5]. Volumetric shrinkage also contributes to these defects, as this occurs when the polymer matrix and the zinc particles contract at different rates during cooling, creating internal stresses within the composite that lead to void formation [4, 5, 24, 33]. The DF images further reveal structural details not visible under BF illumination and confirm the longitudinal homogeneity of zinc dispersion. Up to Zn20, zinc appears well integrated without evidence of clustering, indicating that the extrusion process effectively incorporated the filler. This even distribution is critical for achieving reliable mechanical performance in biomedical applications.

**Figure 4.**
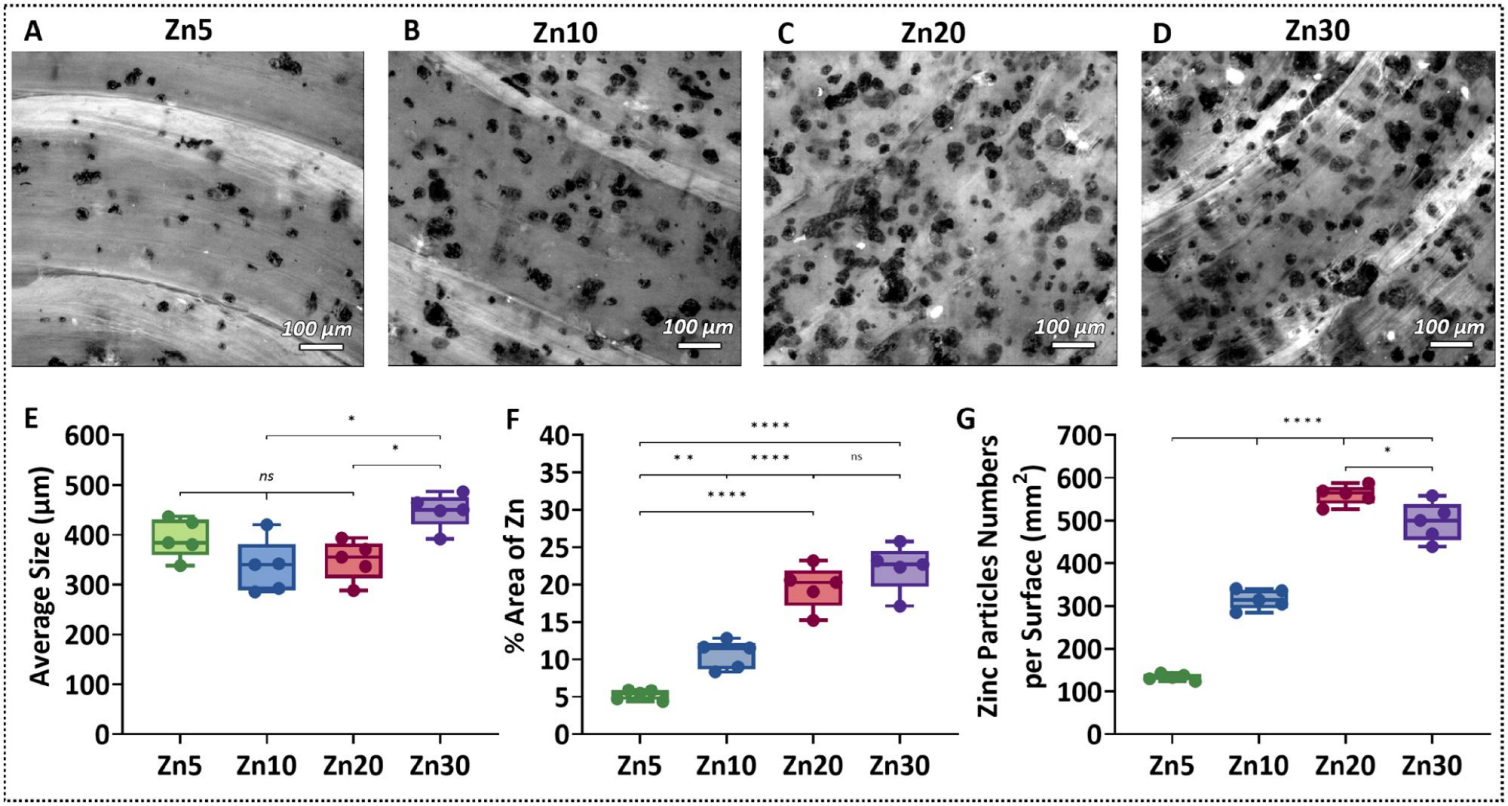
Dispersion and quantification of zinc particles in 3D-printed Zn-reinforced PLA composite. (**A–D**) Optical cross-sections micrographs of Zn5, Zn10, Zn20, and Zn30 showing zinc particle distribution within the PLA matrix respectively. (**E**) Average particle size for each Zn-reinforced PLA composite. (**F**) Surface area fraction occupied by zinc particles. (**G**) Number of zinc particles per unit area. Statistical analysis was performed using one-way ANOVA with Tukey’s honestly significant difference post hoc test. *P < 0.05; **P < 0.01; ****P < 0.0001; ns = not significant.

Figure 4A–D presents the micrograph of the Zn-reinforced PLA composites after the second extrusion during the FDM process. The results show that zinc particles remained well dispersed in the PLA matrix, confirming that printing-induced shear and thermal effects did not lead to significant agglomeration. Figure 4E shows that the average zinc particle size remained statistically consistent across Zn5, Zn10, and Zn20, whereas Zn30 displayed a significant increase in particle size, suggesting particle aggregation at higher filler concentrations. This was further supported by surface area and particle count analysis. As shown in Figure 4F and G, both the percentage of surface area occupied by zinc and the number of particles per unit area increased proportionally from Zn5 to Zn20. However, this trend was disrupted in Zn30, where larger aggregates reduced the number of distinct detectable particles and limited total surface coverage.

These findings were corroborated by density measurements (summarized in Table 2). The density of neat PLA was in close agreement with literature values [43, 44, 67], and the measured densities for Zn5, Zn10, and Zn20 closely matched theoretical values calculated using the rule-of-mixtures. This indicates minimal zinc loss during processing and confirms the successful integration of the metallic phase up to 20 wt%. In Zn30, however, increased porosity and particle clustering resulted in a slight deviation between measured and theoretical density, highlighting a reduction in dispersion efficiency. These findings highlight the inherent challenges of processing high zinc loadings, which lead to clogging, reduced extrudability, and compromise filament flexibility, often resulting in brittle, poorly printable filaments [4–6, 68]. Based on these findings and printing inconsistencies, subsequent testing was focused exclusively on Zn5 and Zn10 composites, while Zn20 and Zn30 were excluded due to their suboptimal processing behaviour.

**Table 2.** Measured and theoretical densities of 3D-printed PLA and Zn-reinforced PLA composite^a^

	Calculated density (g⋅cm^-3^)	Theoretical density (g⋅cm^-3^)
**PLA** ^b^	—	1.24 [43, 44]
**Zinc** ^b^	—	7.14 [5, 46, 47]
**PLA**	1.244 ± 0.007	1.240
**Zn5**	1.291 ± 0.009	1.293
**Zn10**	1.347 ± 0.002	1.352
**Zn20**	1.479 ± 0.008	1.485
**Zn30**	1.48 ± 0.01	1.648

a Measured densities were obtained using Archimedes’ principle, while theoretical densities were calculated using the rule-of-mixtures equation

b Reported in the literature.

### XRD and DSC

**Figure 5.**
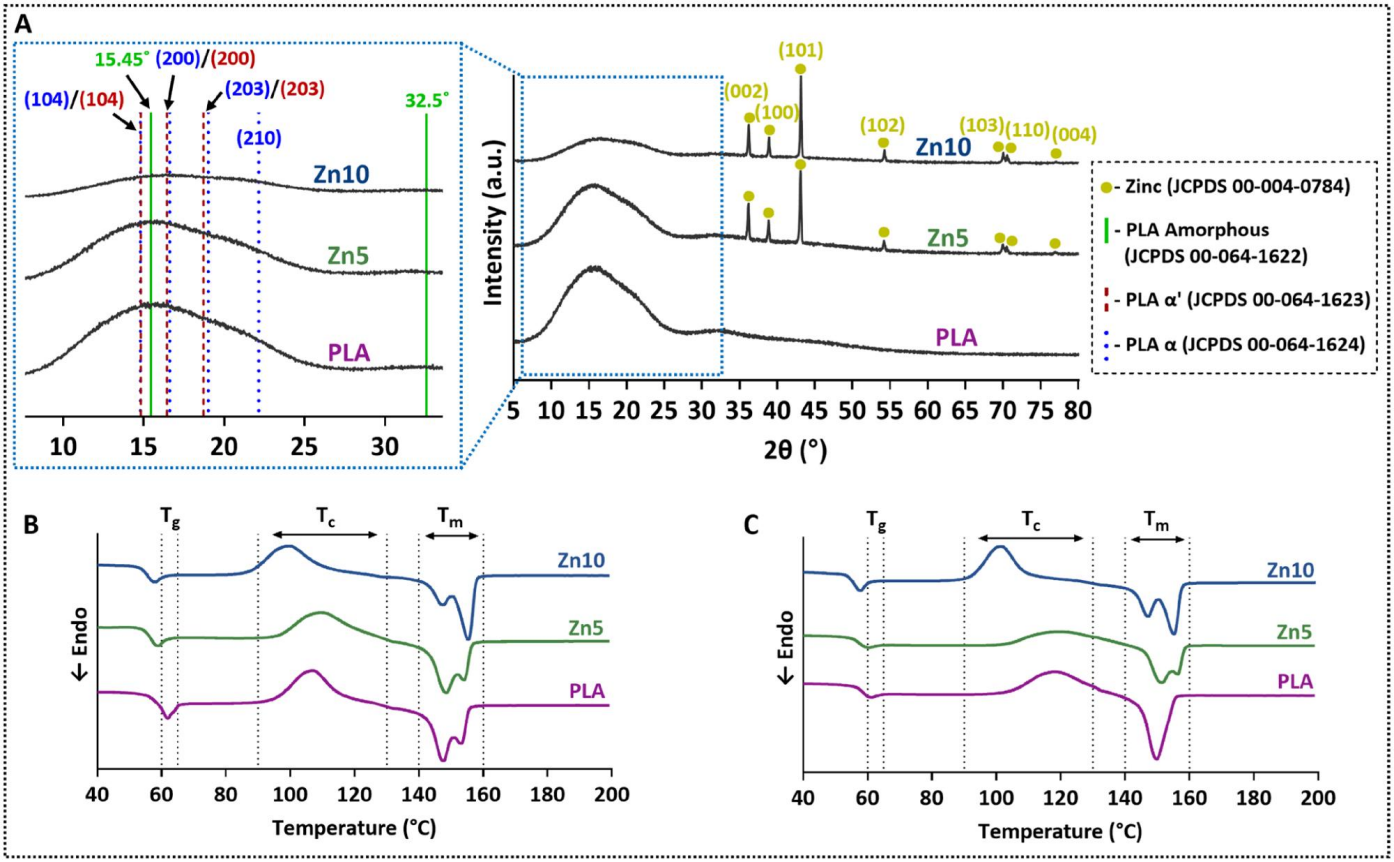
(**A**) XRD diffractogram of PLA, Zn5 and Zn10 3D-printed samples including a magnified section; DSC thermograms of PLA and Zn-reinforced PLA composites during the (**B**) first heating cycle and (**C**) second heating cycle. Each curve illustrates the glass transition (*T*_g_), cold crystallization (*T*_c_), and melting (*T*_m_) events characteristic of the materials.

XRD analysis was performed to investigate the crystallographic structure of neat PLA and Zn-reinforced PLA composites, as shown in Figure 5A. The neat PLA sample exhibited characteristic amorphous and semi-crystalline peaks at 2θ = 15.45° and 32.57°, corresponding to its amorphous phase, in agreement with JCPDS card No. 00-064-1622 [69]. Additionally, crystalline peaks associated with both the α and α′ forms of PLA were identified. Peaks at 14.796°, 16.62°, 19.02°, and 22.30°, corresponding to the (010), (110), (203), and (015) planes, match the α-crystalline phase (JCPDS 00-064-1624), while peaks at 14.819°, 16.436°, and 18.713°, assigned to the same plane families but slightly shifted, are characteristic of the less-ordered α′ phase (JCPDS 00-064-1623) [70]. The presence of both α and α′ phases indicates the coexistence of ordered and disordered crystallites within the PLA matrix, a structural feature that is known to impact its thermal and mechanical properties [11, 12, 71]. In the Zn-reinforced PLA composites, additional sharp diffraction peaks were observed at 36.34°, 39.06°, 43.28°, 54.36°, 70.10°, 70.68°, and 77.04°, which correspond to the (002), (100), (101), (102), (103), (110), and (004) planes of zinc’s hexagonal close-packed (HCP) crystal structure. These reflections are consistent with JCPDS 00-004-0784, confirming the successful incorporation of crystalline Zn particles into the PLA matrix [72]. The calculated unit cell parameters of HCP Zn were a = b ≈ 2.664 Å, c ≈ 4.947 Å, in line with standard literature values, further validating the phase identification and crystallinity of the embedded zinc. The diffraction patterns were explicitly screened for zinc oxide (ZnO). While an ultrathin oxide layer could fall below XRD detection limits, the results indicate no crystalline ZnO formation post-processing.

These findings were also verified by DSC analysis (Figure 5B and C). Across both heating cycles, all samples exhibited two distinct endothermic transitions and one exothermic event, consistent with previously reported PLA-based composites [73]. These features reflect the combined influence of zinc incorporation and the fabrication process on crystallization kinetics and thermal transitions. The first endothermic transition, observed around 53–65°C, corresponds to the glass transition temperature (*T*_g_) of PLA. In the first heating cycle, Zn-reinforced PLA composites exhibited a slightly lower *T*_g_ than neat PLA, potentially due to accelerated chain scission, increased chain-end concentration, or enhanced polymer chain mobility [3, 63, 74–78]. In the second heating cycle, the *T*_g_ values remain relatively consistent compared to the first cycle, demonstrating the intrinsic *T*_g_ after thermal history removal [75, 79, 80]. The broad exothermic peak between 90 and 130°C, attributed to cold crystallization (*T*_c_), reflecting the reorganization of amorphous PLA chains into more ordered crystalline domains upon heating [5, 75, 81]. In the first heating cycle, prominent *T*_c_ peaks in all samples indicate incomplete crystallization during FDM printing, attributed to the rapid cooling rate and inherently slow crystallization kinetics of PLA, which hinder full crystalline development during printing [5]. The *T*_c_ of Zn10 was shifted to lower temperatures compared to PLA and Zn5, suggesting that zinc particles reduced the energy barrier for crystallization initiation [5, 24, 82]. In the second heating cycle, *T*_c_ peaks diminished in neat PLA and Zn-reinforced PLA composites, reflecting reduced recrystallization capacity after melting. For PLA, this aligns with previous reports that amorphous chains lose their ability to reorganize after melt cooling [13, 83]. In Zn5 and Zn10, reduced *T*_c_ peaks likely result from zinc-induced promotion of melt crystallization during cooling [75, 84]. The final endothermic transition, between 140 and 170°C, corresponds to the melting temperature (*T*_m_) of the PLA crystalline regions [9, 10]. Notably, all samples exhibited double melting peaks during the first heating cycle, with zinc addition slightly increasing *T*_m_ [5, 12, 20, 82, 84]. This dual-peak profile, characteristic of heterogeneous crystalline populations, was observed in both neat PLA and Zn-reinforced PLA composites and is indicative of coexisting α and α′ crystalline forms [11, 27]. The first peak is typically associated with the simultaneous melting of less-ordered α′ crystals and the transformation of α′ to α structures, while the second peak corresponds to the melting of more stable α crystals formed during recrystallization [4, 11, 85]. In the second cycle, the double melting peaks disappeared in neat PLA and Zn5 but persisted in Zn10. Their disappearance suggests that the thermal history was erased during the first heating, leading to a more uniform α structure [11, 47]. The persistence in Zn10 indicates ongoing zinc-induced heterogeneous crystallization, producing distinct crystalline domains even after reprocessing [27]. This nucleation effect is supported by the higher crystallinity (*X*_c_) of Zn-reinforced PLA composites, with Zn10 exhibiting the highest *X*_c_, confirming zinc’s role as an efficient nucleating agent that enhances ordered crystalline structure formation and tailors composite thermal properties.

### Mechanical properties

**Figure 6.**
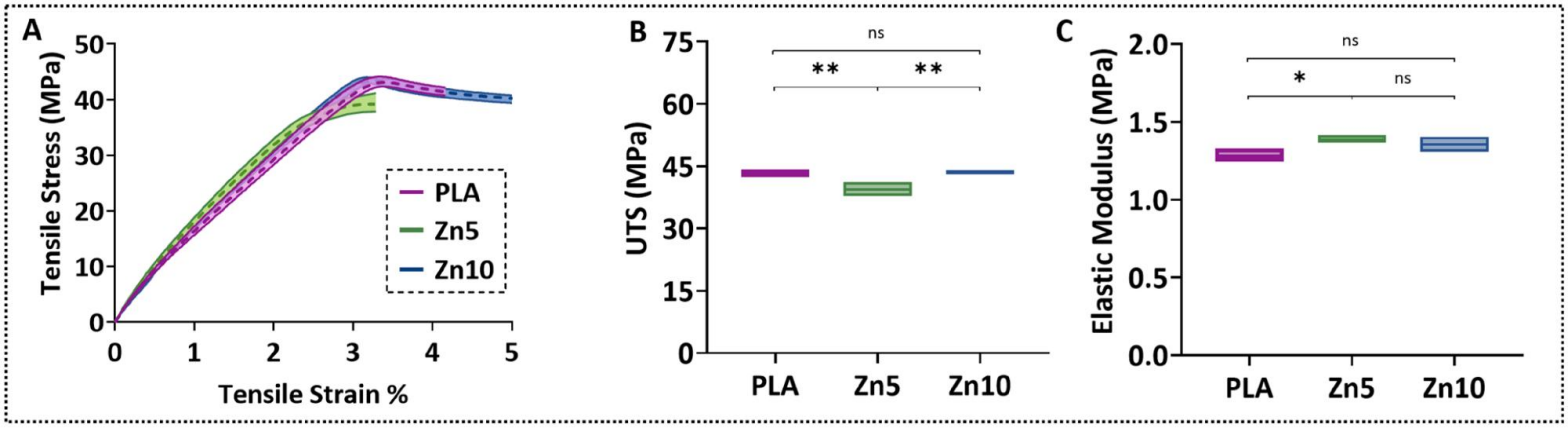
(**A**) Average tensile engineering stress–strain curves of 3D-printed PLA, Zn5, and Zn10 3D-printed samples. (**B**) Ultimate tensile strengths and (**C**) elastic moduli for the same groups. Statistical analysis was performed using one-way ANOVA followed by Tukey’s honestly significant difference post hoc test. *P < 0.05; **P < 0.01; ns = not significant.

Figure 6 presents the average tensile engineering stress–strain curves, ultimate tensile strengths (UTSs), and elastic moduli of 3D-printed PLA, Zn5, and Zn10 samples. The engineering tensile stress–strain curves in Figure 6A show that all samples exhibited typical elastic-plastic deformation [4, 5, 25]. The samples exhibited an initial linear elastic behaviour up to approximately 40 MPa, followed by a nonlinear deformation region leading to the maximum stress. Beyond the peak stress, the stress decreased with increasing strain until failure occurred [86]. The Zn5 composite showed slightly lower tensile strength than neat PLA, whereas Zn10 recovered and marginally exceeded PLA’s performance. This trend is reflected in the UTS values in Figure 6B, where PLA and Zn10 exhibited significantly higher UTS than Zn5, but no significant difference was observed between PLA and Zn10, indicating a partial mechanical recovery at higher Zn loading. Similarly, the elastic modulus values in Figure 6C show a slight increase from PLA to Zn5, followed by no significant difference between Zn5 and Zn10. In terms of ductility, the average strain at fracture was PLA: 4.4 ± 0.3%, Zn5: 3.7 ± 0.5%, and Zn10: 5.5 ± 0.5%, with Zn10 showing the highest strain at fracture, consistent with its recovery in tensile strength.

**Figure 7.**
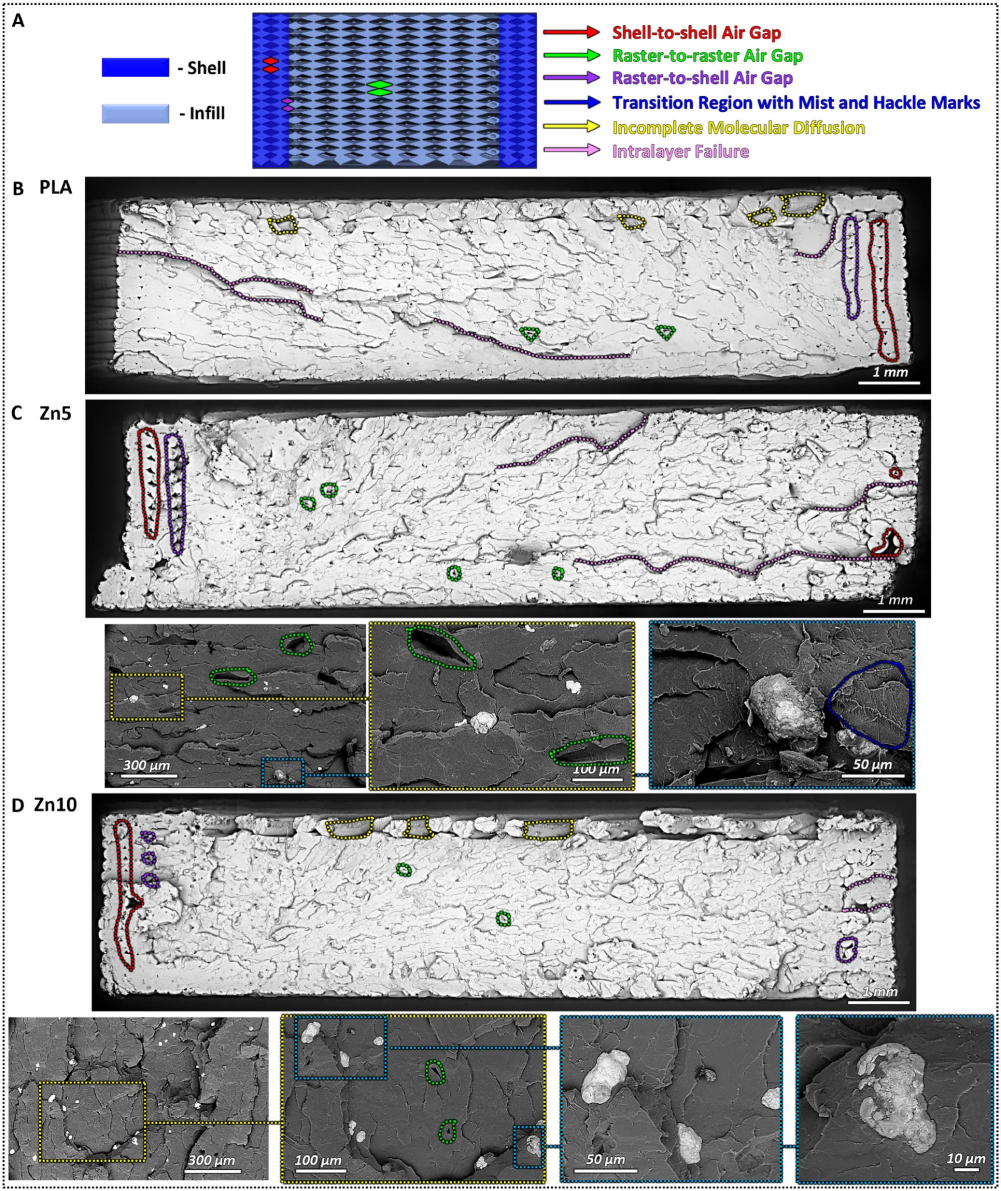
(**A**) Cross-sectional schematic illustrating the printed layer arrangement in the flat orientation, showing the infill pattern (left), and a legend (right) identifying features observed on the fracture surface of the tested specimens. Failure images of post-tensile testing of (**B**) PLA, (**C**) Zn5, and (**D**) Zn10 samples.

Figure 7 presents the fracture surface morphology of the 3D-printed PLA, Zn5, and Zn10 samples after tensile testing. The results of the fracture surface analysis revealed distinct fracture patterns and structural characteristics of the interlayer and intralayer failures [16, 17, 87–89]. All samples exhibited brittle fracture behaviour, consistent with the known fracture characteristics of PLA-based materials under tensile loading [26, 27, 86]. The absence of necking and the relatively smooth fracture surfaces observed are indicative of limited plastic deformation prior to failure [90]. Brittle fracture typically exhibits four major surface regions: (i) mirror, (ii) primary fracture surface, (iii) rough region with Wallner lines, and (iv) transition region with mists and hackle lines [88, 91–93]. Figure 7A provides a digital cross-sectional view of the printed flat sample as generated by the slicing software. This view illustrates the air gaps that inherently form due to the deposition of the printed filament. These air gaps are classified as shell-to-shell (red), raster-to-raster (green), and raster-to-shell (purple). The figure also includes a legend identifying these and other features observed on the fracture surface of the tested specimens. In Figure 7B–D, distinct brittle fracture features such as hackle lines are observed across all printed samples. Furthermore, air gaps, which are unfilled spaces resulting from the deposition algorithm that dictates the spacing between extruded raster lines and layer interfaces, were present in all samples [94, 95]. These air gaps are classified based on their location as shell-to-shell (marked in red), raster-to-shell (marked in purple), and raster-to-raster (marked in green) air gaps. The detrimental effects of air gaps on the mechanical properties of 3D-printed parts are well-documented [96–100]. By reducing interfacial bonding within and between printed layers, they contribute to lower UTS, flexural strength, impact resistance, and stiffness. Additionally, air gaps introduce stress concentration points, which can trigger failure mechanisms such as crazing, delamination, and fracture [97, 101].

The fracture images reveal the characteristic periodic structure of FDM, with a visible raster orientation at ±45° to the loading axis. Across all specimens, triangular-shaped voids were observed between the raster layers, resulting from incomplete filling due to stacking effects inherent to the layer-by-layer FDM process [90]. Strong interlayer fusion was observed across all samples, particularly in the infill regions, where the individual raster lines were no longer distinguishable. This indicates effective bonding between adjacent layers, which contributed to the overall good adhesion. The uniform appearance in these areas suggests that the layer adhesion resulted primarily from sufficient thermal bonding during deposition. In both PLA and Zn10, the triangular air gaps were predominantly located at the shell-to-shell and shell-to-infill interfaces, whereas in Zn5, a greater number of air gaps appeared between infill-to-infill raster lines. Zn5 also exhibited the highest apparent shell-to-shell air gaps, which may correlate with less consistent flow or interlayer bonding during printing. This can explain the lower UTS and ductility that Zn5 showed. Higher-magnification images confirmed that zinc particles were well integrated within the PLA matrix in both Zn5 and Zn10. These particles were uniformly distributed throughout the fracture surface, with no evidence of particle agglomeration, phase separation, or particle pull-out, indicating good compatibility and dispersion within the polymeric matrix [90]. Furthermore, the top printed layers exhibited more pronounced delamination in all samples compared to the infill layers. This can be attributed to differential cooling, as the uppermost layers are exposed to ambient air and experience the highest cooling rates, while the lower layers benefit from the heated build plate (60°C). Optimizing these conditions to improve interlayer bonding is a potential focus for future work, though it is beyond the scope of the current study.

**Figure 8.**
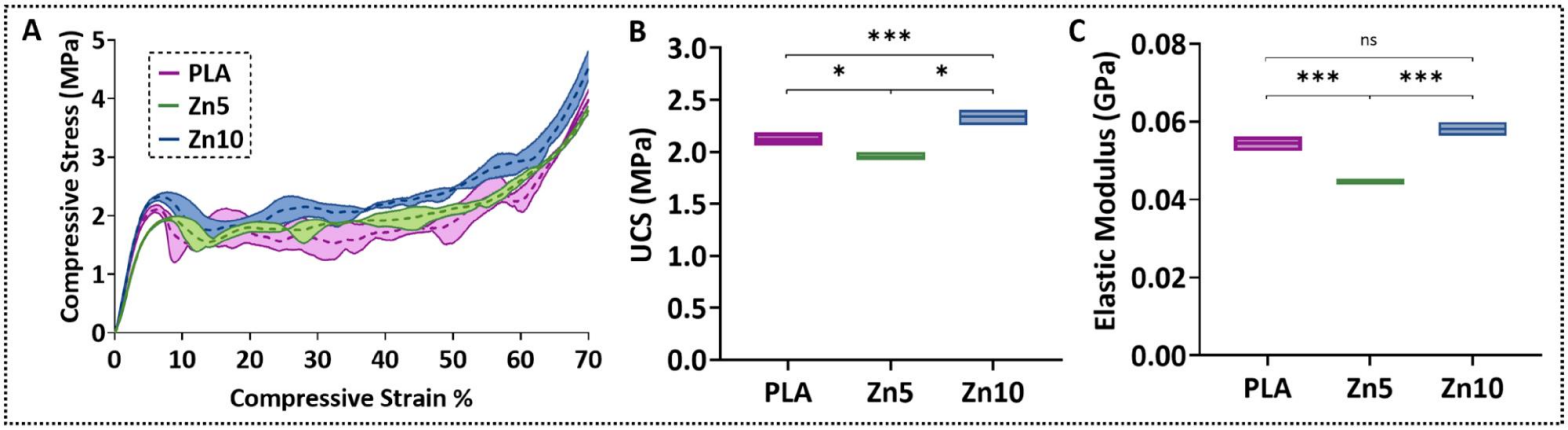
(**A**) Average compression engineering stress–strain curves of 3D-printed PLA, Zn5, and Zn10 specimens featuring a gyroid lattice structure. (**B**) Ultimate compressive strengths and (**C**) elastic moduli for the same groups. Specimens exhibited typical lattice deformation behaviour, including an initial elastic region, a Plateau region of deformation/crushing, and densification at higher strains. Statistical analysis was performed using one-way ANOVA followed by Tukey’s honestly significant difference post hoc test. **P < 0.01; ***P < 0.001; ns = not significant.

Figure 8 presents the average compressive engineering stress–strain curves, ultimate compressive strengths (UCS), and elastic moduli of 3D-printed PLA, Zn5, and Zn10 samples. The gyroid architecture, commonly used in biomimetic scaffolds, is known to produce characteristic stress–strain behaviour typical of cellular structures [45, 102]. This response comprises (i) a linear elastic regime to the first peak, (ii) a plateau governed by cell-wall buckling deformation and brittle crushing, and (iii) densification with a sharp stress rise beyond ∼50% strain. All groups exhibited this pattern (Figure 8A). Quantitatively, Zn10 showed significantly higher UCS and elastic modulus than both PLA and Zn5, whereas Zn5 consistently yielded the lowest values (Figure 8B and C). These compression results align with the tensile trends and indicate that 10 wt% Zn can recover or modestly improve structural performance under compression relative to neat PLA, while 5 wt% Zn underperforms.

The observed decrease in strength and ductility for Zn5 relative to PLA suggests that, at this zinc concentration, detrimental mechanisms dominate, potentially due to the following factors. First, processing-induced molecular weight reduction, potentially exacerbated by the zinc particles, can shift the matrix toward brittle failure, consistent with reports of early crack initiation and damage localization in PLA-Zn systems [5, 75]. Second, even minor particle clustering can act as stress concentrators and locally restrict chain mobility, reducing strain-at-break [75]. Notably, although DSC showed slightly higher crystallinity for Zn5 than PLA, typically favourable for stiffness, this increase was insufficient to offset interfacial defects and the increased brittleness, implicating air gaps and suboptimal stress transfer as the principal causes of performance loss. This trend aligns with the study of Kumar et al. [47] on the PLA-ZnO nanocomposites. A significant drop in tensile strength at low filler concentrations was correlated with a dramatic increase in processing-induced porosity, whereas increasing the filler concentrations reduced porosity and improved mechanical performance. By contrast, Zn10 displayed a recovery in mechanical response versus Zn5, suggesting that beneficial mechanisms emerge at 10 wt% that balance or outweigh the deleterious effects seen at 5 wt%. Potential contributors include: (i) more effective dispersion/reinforcement at 10 wt%, an optimal loading before severe agglomeration undermines properties [103]; (ii) a plasticization-like effect arising from processing-related molecular weight reduction, which can increase chain mobility and flexibility (analogous to enhanced elongation observed with Zn-containing additives) [63, 74, 104]; and (iii) defect minimization during processing that reduces critical air gap density, allowing the improved mobility and particle-matrix interactions to dominate the failure behaviour. Overall, these results underscore the content-dependent influence of Zn on PLA mechanics and help reconcile mixed literature: whereas Pascual-González et al. [5] reported Zn-induced embrittlement and crack acceleration under compression, other metal-filled PLA systems (Mg, Ti, 316L) show property gains with rising filler content [24, 25, 28]. Our findings align more closely with the former, indicating that beyond an optimal threshold, Zn can hinder rather than enhance performance, particularly when dispersion and interfacial adhesion are not fully optimized.

### *In vitro* evaluation

**Figure 9.**
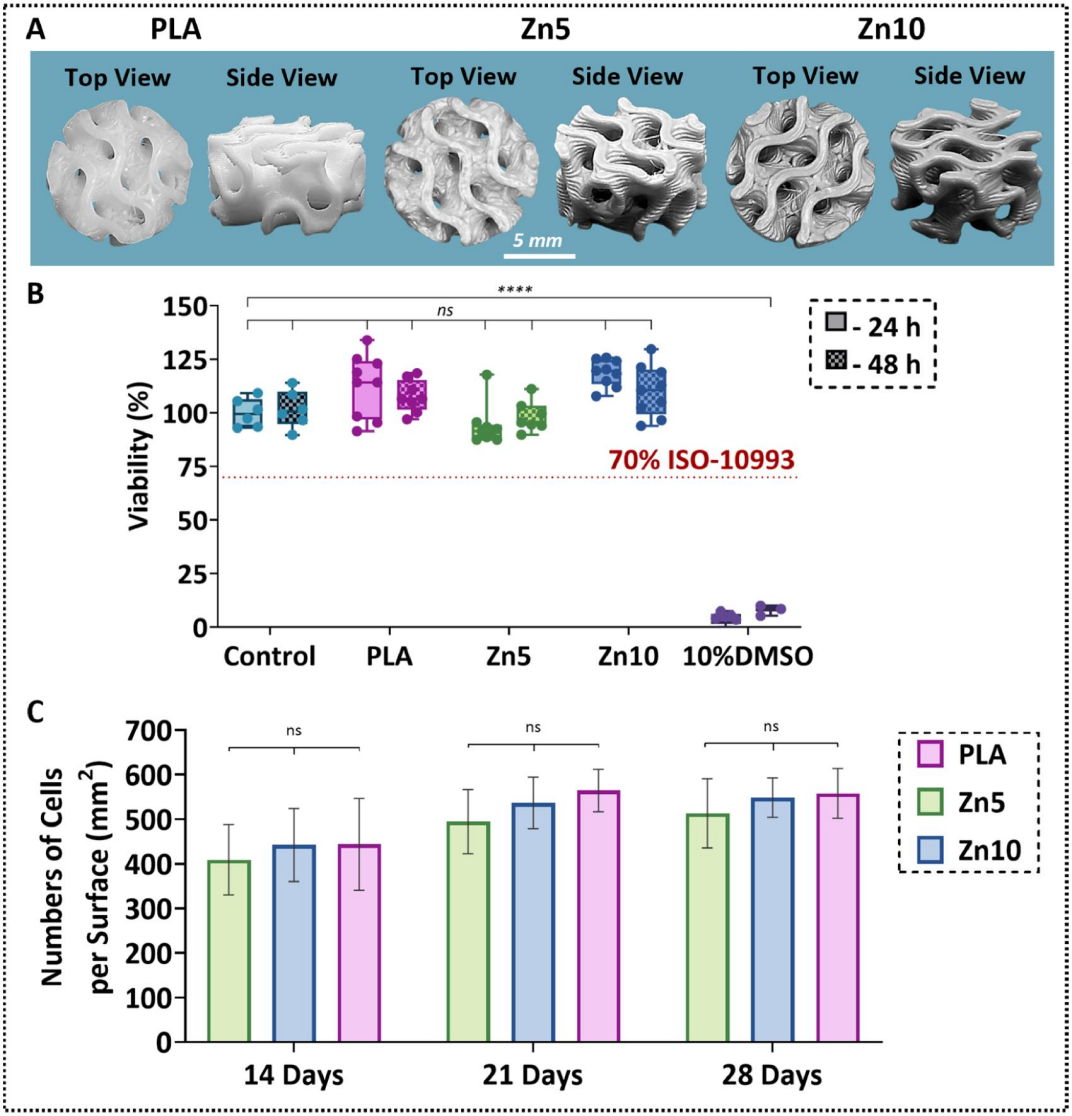
(**A**) Representative images of the 3D-printed gyroid scaffolds used in the *in vitro* evaluation. (**B**) Quantitative results of the indirect cell viability assay of osteoblasts cultured in extract media derived from PLA and PLA-Zinc scaffolds. A 70% viability threshold, as defined by ISO 10993-5, was used to determine biocompatibility. (**C**) Number of cells per field on the scaffolds at each prescribed incubation interval. Statistical analysis was conducted by one-way ANOVA complementary with Tukey’s honestly significant difference test, ****P < 0.0001, ns = no significance.

The biocompatibility and osteogenic potential of 3D-printed PLA, Zn5, and Zn10 gyroid scaffolds (Figure 9A) were assessed using a series of *in vitro* assays, including cytotoxicity testing, cell adhesion quantification, and mineralization analysis. Figure 9B presents the results of the indirect cytotoxicity assay, performed in accordance with ISO 10993-5 standards [57]. Human osteoblasts were exposed to scaffold extract media, and cell viability was quantified relative to a positive control (standard culture medium), with a 10% DMSO-medium mixture serving as a negative control. All tested groups, PLA, Zn5, and Zn10, exhibited cell viability values around 100%, far exceeding the 70% viability threshold defined for non-cytotoxic materials. These results confirm that the Zn-reinforced PLA composites up to 10 wt% zinc are non-cytotoxic to human bone cells [40, 55, 58].

**Figure 10.**
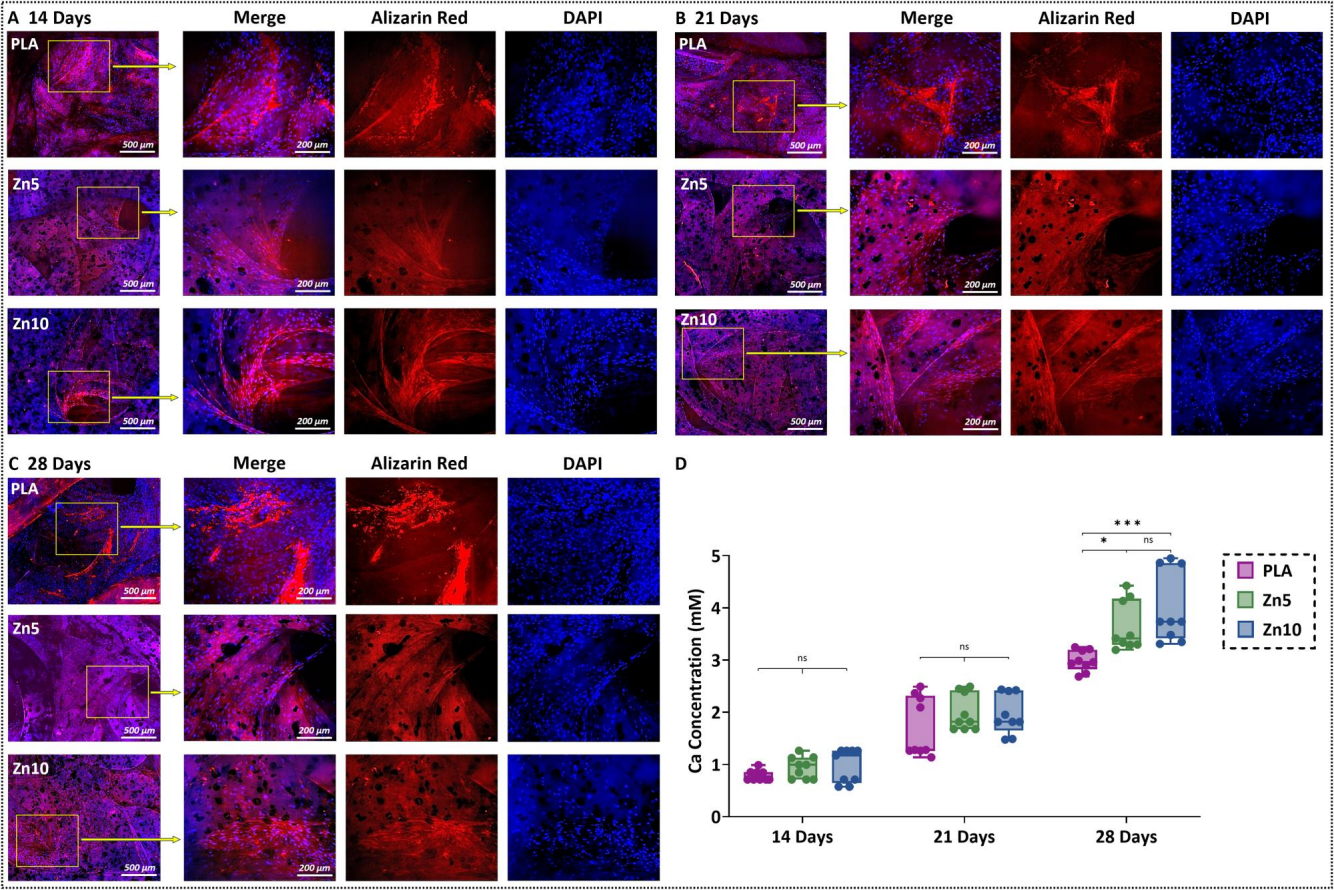
Fluorescence imaging of calcium-rich deposits, stained with alizarin red, of PLA, Zn5, and Zn10 scaffolds on day (**A**) 14, (**B**) 21, and (**C**) 28 of culture. Red areas indicate positive staining for calcium-rich deposits, and blue for DAPI, used to counterstain the nuclei of the fixed cells. (**D**) Calcium concentration at days 14, 21, and 28 for the tested groups was measured from the released alizarin red dye using a plate reader at 405 nm. Statistical analysis was performed using one-way ANOVA followed by Tukey’s honestly significant difference post hoc test. *P < 0.05; ***P < 0.001; ns = not significant.

Cell adhesion, a prerequisite for implant integration and tissue regeneration [59], was assessed by quantifying adherent osteoblast nuclei on scaffold surfaces at days 14, 21, and 28 (Figure 9C and Figure S1). Adherent cell numbers increased over time for all groups, with no statistically significant differences between groups at matched time points. Thus, PLA and Zn-reinforced PLA scaffolds both effectively supported osteoblast attachment and proliferation over the 28-day culture period [59]. To further investigate the surface contributions to the biological response, the wettability on 3D-printed scaffolds was evaluated (Figure S3). The dihedral contact angles did not differ significantly among PLA (73 ± 3°), Zn5 (72 ± 1°), and Zn10 (68 ± 3°), indicating that zinc incorporation did not alter intrinsic surface hydrophilicity. Therefore, the biological outcomes are attributed primarily to the bioactive properties of the zinc, rather than changes in the physical surface characteristic of wettability [3, 74]. Osteogenic differentiation and the mineralization process were examined through fluorescence-based ARS, which visualizes calcium-rich mineral deposits. Representative stained images in Figure 10A–C show the progressive formation of mineralized nodules on all scaffold types from day 14 to day 28. These red fluorescent signals confirm that osteoblasts seeded on both PLA and Zn-reinforced PLA scaffolds underwent osteogenic differentiation, actively depositing mineralized extracellular matrix [40, 55, 58]. Quantitative assessment of calcium deposition is shown in Figure 10D. Across all groups, calcium concentration increased significantly over the 28-day period, reflecting time-dependent mineralization. At days 14 and 21, there were no statistically significant differences between groups, suggesting similar early-stage osteogenic activity [40, 55, 58]. By day 28, however, scaffolds containing zinc exhibited significantly higher calcium levels compared to neat PLA. Zn10 scaffolds demonstrated the highest mineralization overall, although the difference between Zn5 and Zn10 was not statistically significant. These findings indicate that zinc incorporation enhances late-stage osteogenic differentiation, likely due to zinc’s established role in promoting osteoblast maturation and stimulating calcium deposition within the extracellular matrix [35, 36].

In summary, the *in vitro* results confirm that Zn-reinforced PLA scaffolds containing up to 10 wt% zinc are biocompatible, support osteoblast attachment and growth, and significantly promote osteogenic differentiation. These characteristics are critical for materials intended for use in bone tissue engineering. Furthermore, the observed enhancement in mineral deposition may also reflect zinc’s contribution to bioactivity, as discussed in the context of scaffold degradation. While this study provides valuable insights into the behaviour and performance of novel Zn-reinforced PLA composite filaments by establishing the processing window, microstructure-property relationships, and *in vitro* osteogenic response, several limitations merit note. First, comprehensive melt rheology was not performed across the different compositions; hence, future work will quantify viscosity and flow index and correlate these metrics with extrusion behaviour and printability to substantiate the processing data and enhance reproducibility. Second, 3D printing was conducted using parameters optimized for neat PLA, whereas extrusion conditions were composition specific. Accordingly, optimization of printing parameters for each zinc composition will be performed to improve fabrication precision and mechanical performance. Third, porosity was assessed qualitatively and by 2D image inspection only, hence in follow-up studies will include quantitative porosity analysis to resolve 3D pore size distribution, total porosity, and interconnectivity. Fourth, the present work is limited to *in vitro* evaluation. To advance clinical translation, *in vivo* study will be performed to assess long-term performance, including scaffold degradation kinetics, osseointegration, biomechanical fixation, local tissue response, and antibacterial efficacy relevant to post-implantation infection risk. These experiments will place the presented *in vitro* findings in a physiological context and determine whether the observed ion release and bioactivity translate to safe, durable bone regeneration *in vivo*. Despite these limitations, the present work provides a solid foundation for further development and optimization of Zn-reinforced PLA composites for bone tissue engineering.

## Conclusions

This study demonstrates the successful fabrication of zinc-reinforced PLA filaments via melt extrusion and their processing into 3D-printed scaffolds suitable for bone regeneration. Incorporation of up to 10 wt% zinc enabled uniform particle dispersion, preserved mechanical strength, and enhanced scaffold crystallinity. Zn10 scaffolds exhibited mechanical properties comparable to neat PLA, while both Zn5 and Zn10 significantly promoted osteogenic mineralization *in vitro*, underscoring zinc’s role in stimulating late-stage osteoblast differentiation. From a translational perspective, these findings establish zinc-reinforced PLA as a promising, biodegradable composite system for bone tissue engineering. The integration of a bioactive metal within a clinically approved polymer matrix, processed through accessible FDM technology, provides a pathway toward scalable and patient-specific regenerative solutions. Future work will focus on comprehensive melt rheology and thermal transport characterization, optimizing printing parameters, quantitative porosity analysis and *in vivo* investigation to evaluate long-term performance, degradation behaviour, and antibacterial properties. Collectively, this work lays the foundation for advancing multifunctional, extrusion-ready bioactive filaments that meet the demands of next-generation regenerative biomaterials.

## Supplementary Material

rbaf123_Supplementary_Data

## Data Availability

The data that support the findings of this study are available from the corresponding author upon reasonable request.
