# Supplementary material for: Biofabrication of zinc-reinforced PLA scaffolds by FDM for bone tissue engineering
Source: Regen Biomater. 2025 Dec 3;12:rbaf123. doi: 10.1093/rb/rbaf123 (PMC12744395; doi:10.1093/rb/rbaf123)
Supplement: rbaf123_Supplementary_Data [file rbaf123_supplementary_data.zip › Supplementary Materials Accepted - After Fix.docx]

**Supplementary Materials**

**Table S1:** Studies on fabrication and characterization of 3D-printed PLA-composite with metallic additives. Direct Ink Writing (DIW); Fused Deposition Modeling (FDM); Scanning Electron Microscope (SEM); Not Specified (NS); Glass transition temperature (T_g_); Cold crystallization temperature (T_c_); Melting temperature (T_m_); Degree of crystallinity (X_c_). [1–13]

| **System** | **Additive Percentage & Size** | **Extruder** | **AM Printer & Printing Parameters** | **Main Findings** | **Ref** |
| --- | --- | --- | --- | --- | --- |
| PLA-Fe | Fe: 5 wt%  < 150 µm | NS | NS | Adding 5% Fe powder to the PLA matrix significantly improved the mechanical properties, The tensile strength of 5%Fe + PLA was 54.55 MPa compared to 36.46 MPa for neat PLA and Modulus of Elasticity from 644.85 MPa to 686.76 MPa. | [5] |
| PLA-Mg | Mg: 2, 4, 6, 8, and 10 wt%  ≤100 µm | NS | Dayan K12S (FDM)  Nozzle Temp: 210 °C Nozzle Diameter: 0.4 mm Print Speed: 30 mm⋅s^-1^ Layer Height: 0.2 mm Infill Density: 100% Infill Pattern: −45°/+45° | - The fabricated filaments showed a relatively uniform distribution of Mg particles, although some clustering was observed at higher Mg contents (>6 wt%). - Adding Mg particles increased the T_g_ of PLA, suggesting lower mobility of PLA molecular chains due to interference with Mg particles. - Mechanical properties improved up to 8 wt% Mg (33.8% increase in tensile strength and 42% in compressive modulus). - Mg accelerated degradation and improved layer bonding, reducing thermal shrinkage and void formation. | [6] |
| PLA-Mg | Mg: 1.2, 5, 10 and 15 wt%  12.5 ± 7.4 µm | Rondol co-rotating twin-screw extruder and 3devo filament extruder | NS | - The PLA-Mg filaments exhibited significant in vitro biodegradation. - The filaments demonstrated suitable thermal stability for potential 3D printed biomedical applications. | [4] |
| PLA-Ti64 | Ti64: 3,6,9 wt%  10–50 µm | Single Screw Extruder | Dayan K12S (FDM)  Nozzle Temp: 210 °C Print Speed: 35 mm⋅s^-1^ Layer Height: 0.3 mm Infill Density: 60% Infill Pattern: −45°/+45° | - Adding Ti64 increased the T_g_ of PLA. - The addition of 6 wt% Ti64 significantly improved all mechanical properties. The decline in strength at 9 wt% was attributed to agglomerations and voids. | [9] |
| PLA-Mg | Mg: 5, 10, 15, 20 wt%  100 µm | NS | TissueStart 3D bioprinter  (DIW)  Print Speed: 2 mm⋅s^-1^ Layer Height: 0.1 mm Infill Density: 100% | - Mg addition acted as a nucleation agent, speeding up PLA crystallisation. - Thermal stability of PLA decreased with Mg addition. - FTIR analysis suggested potential interaction between Mg particles and the PLA’s ester group. | [3] |
| PLA-Al | Al: 5 wt% | Twin-screw compounder ThemoFisher Scientific | Creality CR-10 S5  FDM  Nozzle Temp: 230, 240, 250 °C Print Speed: 50, 75, 100 mm⋅s^-1^ Layer Height: 0.1, 0.2, 0.3 mm Infill Density: 70, 80, 90% | - A strong bonding between the PLA matrix and the Al reinforcement was observed. - Addition of Al effects the formation of defects and stress concentration areas. - Al powder influences the mechanical properties significantly through its interaction with the matrix. | [13] |
| PLA-Mg | Mg: 1, 2, 3, 4, 5 wt%  <64 µm | Xplore MC 15  3devo Precision 450 | Ultimaker S5 (FDM)  Nozzle Temp: 170-210 °C Print Speed: 5 mm⋅s^-1^ Layer Height: 0.1 mm | - The lowest internal porosity in scaffolds was achieved with PLA and PLA/4% Mg/5% PEG filaments. - Mg particles did not significantly change mechanical properties. - The source highlights that MgO, present in the Mg particles, catalyses the thermal degradation of PLA at extrusion and printing temperatures. - Mg particles promote PLA depolymerisation, triggering degradation at lower temperatures. | [1] |
| Zn-reinforced PLA | Zn: 3.5%, 7%, 10.5%, 14%, and 17.5 wt%  <25 µm | Xplore MC 15  3devo Precision 450 | Ultimaker S5 (FDM)  Nozzle Temp: 170-210 °C Print Speed: 5 mm⋅s^-1^ Layer Height: 0.1 mm | - The double extrusion process successfully filaments with up to 10.5 wt% Zn. - Filaments with higher Zn content showed a significant increase in porosity. - Zn acted as a nucleating agent. - addition of Zn particles resulted in a more brittle failure mode - Higher Zn concentrations could not be printed due to nozzle clogging | [2] |
| PLA-Br/Cu/MI/SS | Br: 80, Cu: 80, MI: 48, SS:8 wt% | NS | Makerbot Replicator 2X, Orion Delta 3-D (FDM)  Nozzle Temp: 200–220 °C Layer Height: 0.1, 0.2, 0.3, 0.4 mm | - High metal vol% significantly lowers PLA mechanical properties. - MI-PLA and SS-PLA with lower metal content perform similarly to neat PLA in tensile and fracture tests. - Br-PLA and Cu-PLA become more ductile, but exhibit decreased ultimate strength and fracture toughness. - Weak metal-PLA adhesion results in particle pull-out and increased porosity. - Metallic particle reinforcement increased the Young's modulus, resulting in a slightly stiffer material. | [11] |
| PLA-316L | 316L: 5,10,15 %v  20–50 µm | EX2, FilaBot | TAZ 6, Lulzbot (FDM)  Nozzle Temp: 205 °C Nozzle Diameter: 0.5 mm Print Speed: 60 mm⋅s^-1^ Layer Height: 0.2 mm Infill Density: 50% Infill Pattern: −90°/+90° | - Mechanical strength improved up to 10 vol% metal content, then declined at 15% due to porosity/agglomeration. - For higher than 10 vol%, voids and powder agglomeration were observed. - 10 vol% had a significantly higher modulus (1.51 GPa) and strength (39.08 MPa) compared to neat PLA (0.22 GPa, 15.73 MPa) and other compositions. - Coefficient of thermal expansion decreased with metal addition, enhancing thermal stability. | [12] |
| PLA-316L/Fe | 316L/Fe: 10 %v  20–50 µm | EX2, FilaBot | TAZ 6, Lulzbot (FDM)  Nozzle Temp: 205 °C Nozzle Diameter : 0.5 mm Print Speed: 60 mm⋅s^-1^ Layer Height: 0.2 mm Infill Density: 100% Infill Pattern: −90°/+90° | - Intra-layer voids and inter-layer gaps were observed in both PLA/316L and PLA/Iron scaffolds. - Both composites showed higher compressive strength and modulus and improved compressive fatigue resistance compared to neat PLA. - Wettability tests revealed neat PLA and PLA/316L surfaces were hydrophobic, while the PLA/Iron surface was hydrophilic. | [10] |
| PLA-Ag | Ag: 4, 9 wt%  50–100 nm | Noztek Pro | Intamsys Funmat HT (FDM)  Nozzle Temp: 230 °C Print Speed: 30 mm⋅s^-1^ Layer Height: 0.1, 0.2, 0.3 mm Infill Density: 100% Infill Pattern: 45° | - 3D-printed parts demonstrated significant antimicrobial properties against bacteria. - Addition of AgNPs resulted in reduced tensile strength compared to neat PLA. - Fracture surface analysis indicated a more ductile behaviour for the nanocomposites compared to the brittle neat PLA. - Microhardness of the nanocomposites was marginally lower than neat PLA. | [8] |
| PLA-Ti | Ti: 0, 6, 10, 16, 20 vol%  23 µm | Single-screw extruder, Jeong-Sung | 3Dison Pro, ROKIT (FDM)  Nozzle Temp: 210 °C Print Speed: 30 mm⋅s^-1^ Layer Height: 0.2 mm Infill Density: 100% | - Homogeneous dispersion of Ti particles in the PLA matrix was achieved at loadings of 5–15 vol%. Agglomeration occurred at 20 vol%. - T_g_ and T_m_ slightly increased with increasing Ti loading, T_c_ and X_c_ of PLA decreased. - In vitro biocompatibility was enhanced by the incorporation of Ti. | [7] |

## Particle Size Analysis

The morphology and particle-size distribution of the zinc powder were characterized prior to filament fabrication. Powders were imaged by SEM (Phenom XL, Thermo Fisher Scientific, Eindhoven, The Netherlands) to obtain high-resolution micrographs. Images were analyzed in FIJI (ImageJ): after brightness/contrast adjustment and thresholding to generate binary masks, the Analyze Particles tool was used to compute the equivalent circular diameter for each particle. The analysis (n > 300 particles) yielded an average particle size of 66 ± 28 µm, in close agreement with the manufacturer’s specification (~ 70 µm). The results show that the zinc particles exhibit irregular, non-spherical morphologies with a relatively broad size distribution, consistent with the SEM micrographs (Figure S1). Using non-spherical zinc powder can be advantageous for PLA composites intended for bioresorbable scaffolds. Irregular particles provide a higher surface area-to-volume ratio than spheres, which can accelerate polymer degradation pathways and promote faster resorption of the construct [6]. From a mechanics standpoint, anisometric fillers (e.g., rod-like or fibrous morphologies) can offer more effective reinforcement than spherical particles by improving stress transfer and hindering crack propagation [14]. At small scales, spherical nanoparticles are also more prone to agglomeration due to high surface energy, reducing their effective interfacial area and masking geometry effects; irregular morphologies may disperse more favourably and preserve active surface area [15–17]. Finally, for antibacterial functionality, several studies report higher biocidal activity for non-spherical ZnO architectures (e.g., wires/rods/“flower” shapes) relative to spheres, potentially enhancing anti-infective performance of the scaffold surface [16]. Taken together, these considerations support selecting non-spherical zinc morphologies to balance degradation kinetics, mechanical reinforcement, dispersion stability, and antibacterial efficacy in PLA-based composites.


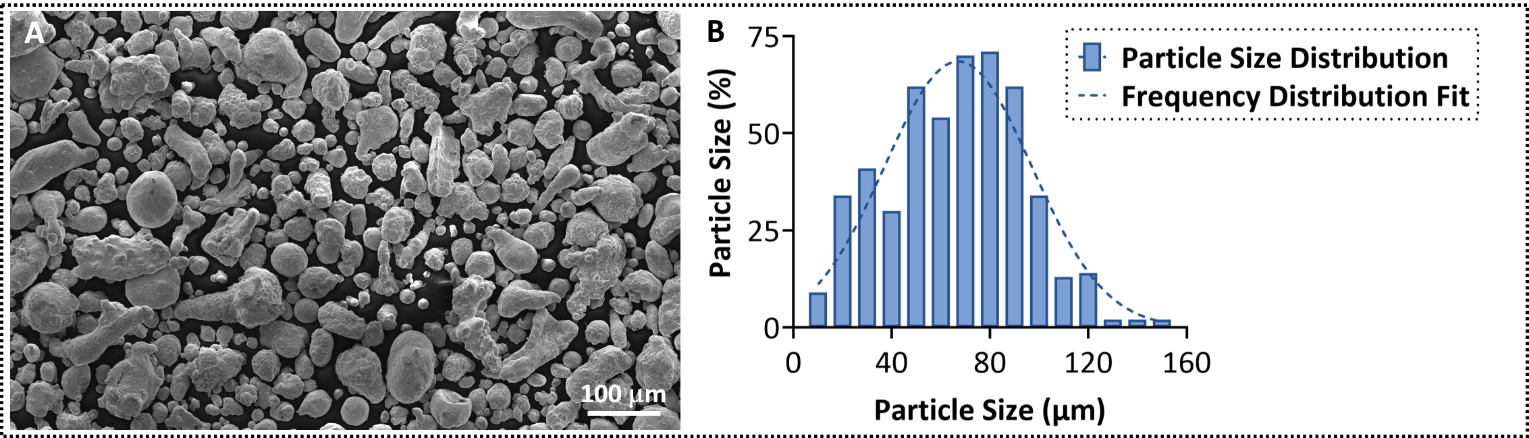


**Figure S1.** Characterization of the zinc powder. **(A)** Representative SEM micrograph showing the irregular morphology of the zinc particles. **(B)** Particle size distribution histogram obtained from image analysis, with a frequency distribution fit indicating a peak around 70-80 µm.

## In vitro Additions


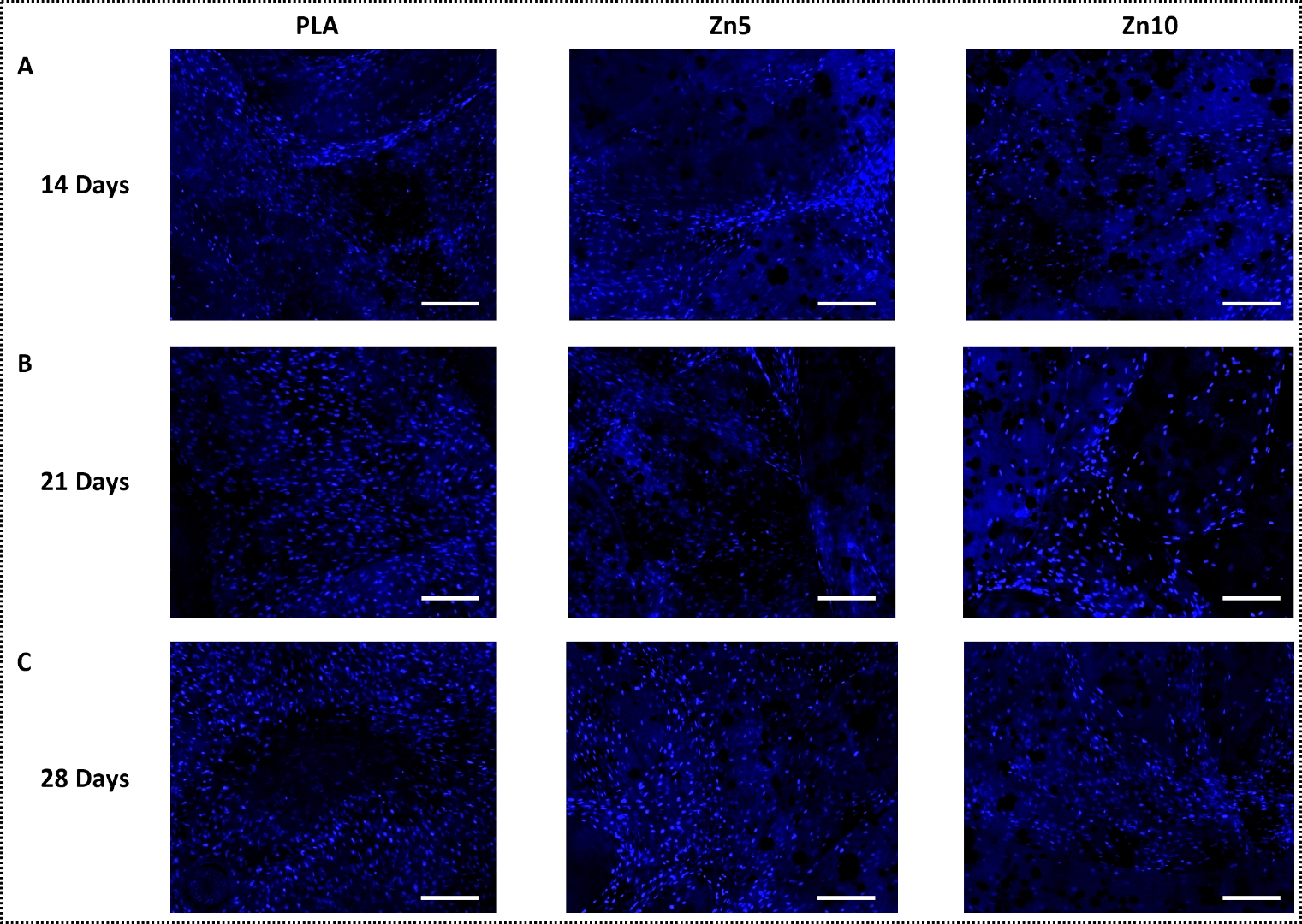


**Figure S2.** Fluorescence images of osteoblasts stained with DAPI that were attached on the surface of PLA, Zn5, and Zn10 at **(A)** 14 days, **(B)** 21 days, and **(C)** 28 days of incubation (scale bar: 200 µm).

#### Surface wettability test

Surface wettability was evaluated by static sessile-drop contact angle measurements. Flat coupons of neat PLA, Zn5, and Zn10 were printed with 100% rectilinear infill to produce a smooth, planar top surface. For each specimen, three 20 µL droplets of Minimum Essential Medium Alpha (Sartorius, Germany) were gently dispensed onto the surface, and droplet images were captured with a digital camera. Contact angles were extracted using the LB-ADSA (Low-Bond Axisymmetric Drop Shape Analysis) plugin in FIJI/ImageJ [18]. The results show that contact angle measurements had no statistically significant differences in surface wettability between the neat PLA, Zn5, and Zn10 groups. This suggests that the incorporation of up to 10 wt% zinc does not substantially alter the intrinsic hydrophilicity of the 3D-printed PLA surface.


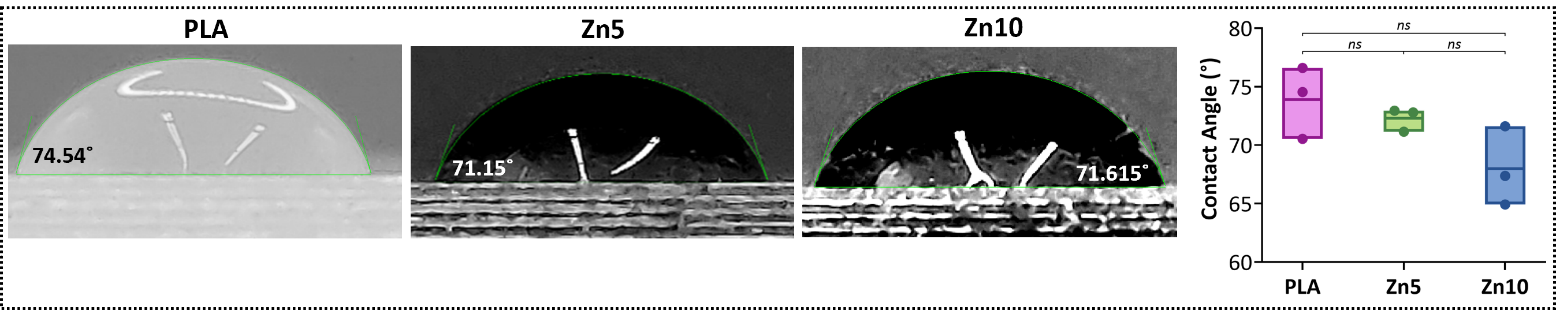


**Figure S3.** Contact angle analysis of Zn-reinforced PLA composites. **(A)** Sessile drop images showing the contact angle of cell culture medium on the surfaces of 3D-printed PLA, Zn5, and Zn10 coupons. **(B)** A graph summarizing the average contact angles and the results of a one-way ANOVA, which show no statistically significant differences in wettability among the tested materials.

## References

[1] C. Pascual-González, C. Thompson, J. de la Vega, N. Biurrun Churruca, J.P. Fernández-Blázquez, I. Lizarralde, D. Herráez-Molinero, C. González, J. LLorca, Processing and properties of PLA/Mg filaments for 3D printing of scaffolds for biomedical applications, Rapid Prototyp. J. 28 (2021) 884–894. https://doi.org/10.1108/RPJ-06-2021-0152.

[2] C. Pascual-González, J. de la Vega, C. Thompson, J.P. Fernández-Blázquez, D. Herráez-Molinero, N. Biurrun, I. Lizarralde, J.S. del Río, C. González, J. LLorca, Processing and mechanical properties of novel biodegradable poly-lactic acid/Zn 3D printed scaffolds for application in tissue regeneration, J. Mech. Behav. Biomed. Mater. 132 (2022) 105290. https://doi.org/10.1016/j.jmbbm.2022.105290.

[3] F. Ali, A. Al Rashid, S.N. Kalva, M. Koç, Mg-Doped PLA Composite as a Potential Material for Tissue Engineering—Synthesis, Characterization, and Additive Manufacturing, Materials 16 (2023) 6506. https://doi.org/10.3390/ma16196506.

[4] A. Leonés, V. Salaris, I. Ramos Aranda, M. Lieblich, D. López, L. Peponi, Thermal Properties and In Vitro Biodegradation of PLA-Mg Filaments for Fused Deposition Modeling, Polymers 15 (2023) 1907. https://doi.org/10.3390/polym15081907.

[5] M. Memiş, D.A. Gök, Development of Fe-reinforced PLA-based composite filament for 3D printing: Process parameters, mechanical and microstructural characterization, Ain Shams Eng. J. 16 (2025) 103279. https://doi.org/10.1016/j.asej.2025.103279.

[6] R. Bakhshi, M. Mohammadi-Zerankeshi, M. Mehrabi-Dehdezi, R. Alizadeh, S. Labbaf, P. Abachi, Additive manufacturing of PLA-Mg composite scaffolds for hard tissue engineering applications, J. Mech. Behav. Biomed. Mater. 138 (2023) 105655. https://doi.org/10.1016/j.jmbbm.2023.105655.

[7] J. Lee, H. Lee, K.H. Cheon, C. Park, T.S. Jang, H.E. Kim, H. Do Jung, Fabrication of poly(lactic acid)/Ti composite scaffolds with enhanced mechanical properties and biocompatibility via fused filament fabrication (FFF)–based 3D printing, Addit. Manuf. 30 (2019) 100883. https://doi.org/10.1016/j.addma.2019.100883.

[8] N. Vidakis, M. Petousis, E. Velidakis, M. Liebscher, L. Tzounis, Three-Dimensional Printed Antimicrobial Objects of Polylactic Acid (PLA)-Silver Nanoparticle Nanocomposite Filaments Produced by an In-Situ Reduction Reactive Melt Mixing Process, Biomimetics 5 (2020) 42. https://doi.org/10.3390/biomimetics5030042.

[9] M. Zarei, M. Shabani Dargah, M. Hasanzadeh Azar, R. Alizadeh, F.S. Mahdavi, S.S. Sayedain, A. Kaviani, M. Asadollahi, M. Azami, N. Beheshtizadeh, Enhanced bone tissue regeneration using a 3D-printed poly(lactic acid)/Ti6Al4V composite scaffold with plasma treatment modification, Sci. Rep. 13 (2023) 3139. https://doi.org/10.1038/s41598-023-30300-z.

[10] D. Jiang, F. Ning, Fused filament fabrication of biodegradable PLA/316L composite scaffolds: Effects of metal particle content, Procedia Manuf. 48 (2020) 755–762. https://doi.org/10.1016/j.promfg.2020.05.110.

[11] V.S. Vakharia, L. Kuentz, A. Salem, M.C. Halbig, J.A. Salem, M. Singh, Additive Manufacturing and Characterization of Metal Particulate Reinforced Polylactic Acid (PLA) Polymer Composites, Polymers 13 (2021) 3545. https://doi.org/10.3390/polym13203545.

[12] D. Jiang, F. Ning, Y. Wang, Additive manufacturing of biodegradable iron-based particle reinforced polylactic acid composite scaffolds for tissue engineering, J. Mater. Process. Technol. 289 (2021) 116952. https://doi.org/10.1016/j.jmatprotec.2020.116952.

[13] K.R. Kumar, M. Gokul, M. Kumar, Investigations on Mechanical Properties and Characterisation of Polylactic Acid/Aluminium Metal Infill Polymer Composites Manufactured by Fused Deposition Modelling, J. Mater. Eng. Perform. 33 (2024) 8857–8870. https://doi.org/10.1007/s11665-023-08572-3.

[14] I. Plamadiala, C. Croitoru, M.A. Pop, I.C. Roata, Enhancing Polylactic Acid (PLA) Performance: A Review of Additives in Fused Deposition Modelling (FDM) Filaments, Polymers 17 (2025) 191. https://doi.org/10.3390/polym17020191.

[15] W.J. Chong, S. Shen, Y. Li, A. Trinchi, D. Pejak, I. (Louis) Kyratzis, A. Sola, C. Wen, Additive manufacturing of antibacterial PLA-ZnO nanocomposites: Benefits, limitations and open challenges, J. Mater. Sci. Technol. 111 (2022) 120–151. https://doi.org/10.1016/j.jmst.2021.09.039.

[16] B.K. Dejene, Reviewing the manufacturing challenges and scientific debates: Insights into the antibacterial capabilities and potential applications of PLA/ZnO nanocomposites, J. Thermoplast. Compos. Mater. 38 (2025) 2779–2849. https://doi.org/10.1177/08927057241292298.

[17] O.A. Serenko, V.I. Roldughin, A.А. Askadskii, E.S. Serkova, P.V. Strashnov, Z.B. Shifrina, The effect of size and concentration of nanoparticles on the glass transition temperature of polymer nanocomposites, RSC Adv. 7 (2017) 50113–50120. https://doi.org/10.1039/C7RA08152A.

[18] A.F. Stalder, T. Melchior, M. Müller, D. Sage, T. Blu, M. Unser, Low-bond axisymmetric drop shape analysis for surface tension and contact angle measurements of sessile drops, Colloids Surf. Physicochem. Eng. Asp. 364 (2010) 72–81. https://doi.org/10.1016/j.colsurfa.2010.04.040.
